# Regulation of exocytosis and mitochondrial relocalization by Alpha-synuclein in a mammalian cell model

**DOI:** 10.1038/s41531-019-0084-6

**Published:** 2019-06-27

**Authors:** Meraj Ramezani, Marcus M. Wilkes, Tapojyoti Das, David Holowka, David Eliezer, Barbara Baird

**Affiliations:** 1 Department of Chemistry and Chemical Biology, Cornell University, Ithaca, NY 14853 USA; 2 Department of Biochemistry, Weill Cornell Medicine, New York, NY 10065 USA

**Keywords:** Microscopy, Cell biology, Cellular neuroscience, Parkinson's disease

## Abstract

We characterized phenotypes in RBL-2H3 mast cells transfected with human alpha synuclein (a-syn) using stimulated exocytosis of recycling endosomes as a proxy for similar activities of synaptic vesicles in neurons. We found that low expression of a-syn inhibits stimulated exocytosis and that higher expression causes slight enhancement. NMR measurements of membrane interactions correlate with these functional effects: they are eliminated differentially by mutants that perturb helical structure in the helix 1 (A30P) or NAC/helix-2 (V70P) regions of membrane-bound a-syn, but not by other PD-associated mutants or C-terminal truncation. We further found that a-syn (but not A30P or V70P mutants) associates weakly with mitochondria, but this association increases markedly under conditions of cellular stress. These results highlight the importance of specific structural features of a-syn in regulating vesicle release, and point to a potential role for a-syn in perturbing mitochondrial function under pathological conditions.

## Introduction

Parkinson’s disease (PD) is the second most common neurodegenerative disorder.^1^ With increased risk of diagnosis after age 60, PD onset strongly correlates with age, affecting ~1–2% of the population over age 65.^2^ Clinically, motor dysfunction in PD is associated with death of dopaminergic neurons in the substantia nigra, coupled with the presence of abnormal protein aggregates, known as Lewy bodies, in surviving neurons.^2,3^ The primary component of these intraneuronal aggregates is the presynaptic protein alpha synuclein (a-syn).^4^ A-syn inclusions are also a defining feature of Parkinson’s Disease Dementia and Lewy Body Dementia, as well as a common co-pathology in Alzheimer’s disease.^5^

First identified as binding to synaptic vesicles,^6^ a-syn has been characterized as a 140 amino acid, intrinsically disordered protein found predominantly in neurons.^7^ A-syn is genetically linked to autosomal dominant early onset familial PD through point mutations and gene duplication or triplication.^8–10^ Elevated levels of a-syn are observed in many patients who develop sporadic forms of PD, suggesting a critical role for this protein in the majority of PD cases.^11,12^ Moreover, genome wide-association studies have identified the gene that encodes for a-syn, SNCA, as one of the strongest risk loci for sporadic forms of PD.^13^ A-syn has been the subject of intense research with the aim of understanding the relationship between a-syn physiological function and PD pathology, but this connection remains poorly understood at the cellular level.

**Fig. 1:**
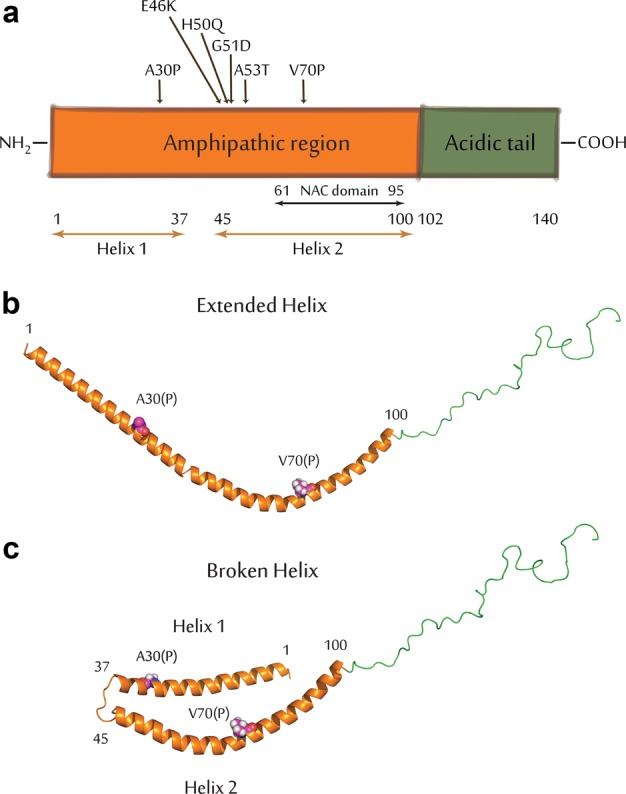
Structural features of a-syn. **a** Schematic representation of the a-syn primary sequence delineating the amphipathic membrane-binding domain (orange) and the acidic C-terminal tail (green), and indicating the locations of the NAC domain, helix-1 and helix-2 of the broken-helix state, and the sites of PD and other mutations examined in the manuscript. **b** Cartoon model of Wt a-syn in an extended helix conformation. The model was generated from RCSB protein data bank entry 1XQ8 by manually converting the non-helical linker between helix-1 and helix-2 to a helical conformation. The sidechains of residues Ala 30 and Val 70 are shown to indicate the location of proline mutations examined in this study. **c** Wt a-syn in the broken helix conformation (RCSB protein data bank entry 1XQ8) with the sidechains of Ala 30 and Val 70 shown

Several studies with a-syn in neuronal cells and synaptosomes derived from rodent brains implicate a critical role in regulation of SV trafficking (exocytosis and endocytosis) and homeostasis,^14^ and membrane binding properties related to a-syn structure have been evaluated in vitro.^15,16^ The amphipathic N-terminal segment (residues 1–100; Fig. 1a) forms an extended helix upon binding to negatively charged phospholipid vesicles (Fig. 1b), which breaks into two smaller helices when binding to phospholipid micelles (Fig. 1c). The broken helix form has been proposed to resemble the structure of a-syn when binding simultaneously to two phospholipid membranes, such as bridging a synaptic vesicle with the plasma membrane.^17^ The acidic C-terminal segment of a-syn (residues 100–140) is disordered and suppresses a-syn aggregation mediated by the N-terminal NAC region (Fig. 1a).

Delineating the structural mechanisms by which a-syn regulates SV trafficking, and if and how this becomes dysregulated, is necessary for understanding PD onset and pathology. Toward this objective, we developed an experimental system based on trafficking and stimulated exocytosis of recycling endosomes (REs) in RBL-2H3 mast cells, which have proven useful as a model system for investigating general questions of membrane and cell biology. RBL cell responses are typically triggered via antigen crosslinking of immunoglobulin E bound to its high affinity receptor (IgE/FcεRI), which results in exocytosis of both secretory lysosomes (degranulation) and recycling endosomes (REs).^18,19^ However, just as neuronal release of SVs involves increase of cytosolic Ca^2+^ concentration,^20^ so does stimulated exocytosis in RBL cells involve Ca^2+^ mobilization that can be triggered directly with thapsigargin, which inhibits the sarco/endoplasmic Ca^2+^ ATPase.^21^ Also consistent with this approach, a previous study suggested that a-syn inhibition of dopamine exocytosis in PC12 and chromaffin cells occurs downstream of stimulated Ca^2+^ responses.^22^

As described herein, our studies demonstrated a clear phenotype: Human a-syn expressed at low levels in RBL cells inhibits stimulated exocytosis of REs. With this result as a starting point we systematically compared Wt a-syn to a panel of a-syn variants, including a-syn mutants associated with PD and a-syn with mutations in N-terminal helical and C-terminal tail regions (Fig. 1). We correlated functional effects with effects on binding modes to model membranes in vitro, quantified using nuclear magnetic resonance (NMR) measurements. Surprisingly, we found that whereas low expression levels of Wt a-syn inhibit stimulated RE exocytosis, high expression levels have an enhancing effect. Moreover, the effects of specific a-syn mutants are different for high vs. low expression levels. These differential effects prompted us to evaluate intracellular distributions of a-syn and REs using fluorescence imaging, and we observed that high level expression of a-syn causes dispersal of REs from the endocytic recycling compartment (ERC), which may play a role similar to the reserve pool of SVs in neurons. We also observed a-syn association with mitochondria, depending on expression level, mutations, and mitochondrial stress. Mitochondrial dysfunction and stress are strongly implicated in PD by genetics (e.g., PINK/parkin pathway) and environmental factors (e.g., pesticides), and stress-dependent association of a-syn with mitochondria could provide a link between these two different critical pathways in PD.

Overall, our results provide a self-consistent picture of how a-syn affects the functions of RBL cells that can be related directly to modes of a-syn binding to synthetic membranes as measured with NMR. The picture emerging for a-syn effects is that stimulated RE exocytosis is: 1) inhibited by high affinity binding to docked vesicles via a broken helix, requiring an intact helix 2 (Fig. 1c); and 2) enhanced at high expression levels by curvature sensitive, low affinity binding to isolated vesicles/tubules involving helix 1 (Fig. 1b), leading to increased availability of vesicles for docking. Specific membrane binding properties also provide insight to variable association of Wt a-syn and mutants with mitochondria with and without stress. We point to the parallels between trafficking of endosomal vesicles and association with internal organelles in neurons and non-neuronal cells, and suggest that the a-syn interactions and effects we have characterized with REs in mast cells may be related to the interactions of a-syn with SVs and mitochondria within neurons that are implicated in the etiology of PD.

## Results

**Fig. 2:**
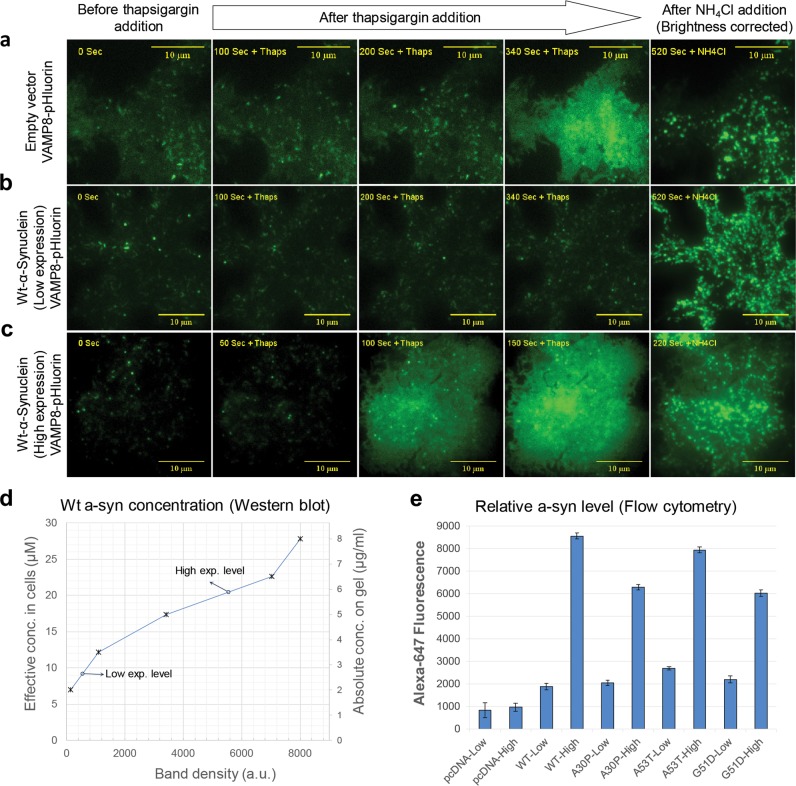
Distinct expression levels of Wt a-syn show differential effects on stimulated exocytosis of REs. **a**–**c** Snapshots from representative movies taken with TIRFM (Supplementary Movies 1a–c); scale bar = 10 μm. As indicated, RBL cells were co-transfected with VAMP8-pHluorin and empty plasmid pcDNA **a** or low (5 μg **b**) or high (25 μg **c**) levels of this plasmid containing Wt a-syn. Exocytosis was stimulated by thapsigargin, and VAMP8-pHluorin fluorescence increase was monitored before and after stimulation, and after addition of NH_4_Cl (50 mM, 300–400 s later) to dequench intracellular VAMP8-pHluorin fluorescence. Brightness of images in right-most column was reduced by 50% for better detail visualization. **d** Concentration of Wt a-syn expressed in RBL cells transfected with low (5 μg) or and high (25 μg) levels of plasmid containing this construct, analyzed from representative western blot shown in Supplementary Fig. 1. All samples in this blot were derived from the same experiment, processed in parallel, and run on the same gel. A calibration curve of band density vs. absolute concentration (right *y*-axis) was determined for purified Wt a-syn (ӿ). Absolute concentrations of Wt a-syn in transfected cells (o) were determined from the calibration curve after normalizing a-syn band densities with respect to nonspecific bands used as a loading control. The corresponding concentration in RBL cells at low (9 μM) and high (20 μM) expression levels of Wt a-syn was then calculated after accounting for number of cells and cell volume as described in Materials and methods section. **e** Cells co-transfected with VAMP8-pHluorin and pcDNA, Wt a-syn or a-syn mutants at low (5 μg) or high (25 μg) levels were fixed, immunostained with anti-a-syn and Alexa 647, and analyzed using flow cytometry. Alexa 647 fluorescence histograms were measured for RBL cells gated by VAMP8-pHluorin fluorescence (see Supplementary Fig. 2). Averaged peak values of histograms from three independent experiments, with 15,000–20,000 total VAMP8-pHluorin labeled cells were analyzed for each sample type shown. Error bars are coefficients of variance

*Wt a-syn interferes with stimulated exocytosis in RBL cells*. We showed previously that both antigen and thapsigargin stimulate exocytosis of recycling endosomes (REs),^23^ and we evaluated this process as a proxy for a similar process occurring with synaptic vesicles (SVs) in neurons. To visualize stimulated RE exocytosis by microscopy, RBL cells are transfected with DNA for the reporter VAMP8-pHluorin, a v-SNARE protein conjugated to a fluorophore that is quenched when localized to the mildly acidic environment of intracellular REs. As shown vividly by TIRF microscopy for cells co-transfected with an empty vector (pcDNA) and stimulated with thapsigargin, exocytotic events appear as increases in VAMP8-pHluorin fluorescence at the plasma membrane where REs are exposed to the neutral pH environment of the extracellular medium (Fig. 2a, Supplementary Movie 1a).^23^ Addition of NH_4_^+^Cl^-^ at the end of the time course neutralizes the pH in all cellular compartments and provides a means of normalizing the level of the stimulated exocytosis. We found that cells expressing low levels of Wt a-syn exhibit highly inhibited thapsigargin-stimulated exocytosis (Fig. 2b and Supplementary Movie 1b). In contrast, cells transfected with the same amount of DNA for VAMP8-pHluorin, but with five times the amount of DNA for Wt a-syn, exhibit a greater extent of exocytosis compared to pcDNA controls (Fig. 2c and Supplementary Movie 1c). As described in following sections, we quantified the contrasting effects of a-syn at lower and higher expression levels on multiple cellular processes, and we evaluated selected a-syn mutants for additional insight into the structural interactions involved. Table 1 summarizes key results.

**Table 1:** Functional effects and structural interactions of Wt a-syn and selected mutants

Conditions	Wt-a-syn		A30P-a-syn		V70P-a-syn	
Stimulated exocytosis—low expression (Fig. 2f)	↓+++		↓+++		–	
Stimulated exocytosis—high expression (Fig. 2h)	↑+		↓+++		↑+	
Binding to mitochondria—no stress (Fig. 6)	+		–		–	
Binding to mitochondria—with stress (Fig. 6)	+++		+		–	
Binding to lipid droplets—high expression levels (Fig. S6)	+++		–		NT	
Disruption of vesicle binding (SUVs, NMR) (Fig. 3, S3)	Helix1	Helix2	Helix1	Helix2	Helix1	Helix2
	–	–	+	+++	–	+++
Disruption of secondary structure (micelles, NMR) (Fig. 3)	Helix1	Helix2	Helix1	Helix2	Helix1	Helix2
	–	–	+	–	–	+

*↑ and ↓ indicate enhancing or inhibitory effect, respectively; +++, high effect; +, low effect; –, no effect; NT, not tested

*Low and high expression levels of human a-syn are quantified*. We directly compared expression levels corresponding to conditions of Fig. 2b, c by immunostaining with monoclonal human a-syn antibody. We detected no a-syn labeling above background in pcDNA transfected cells (Supplementary Fig. 1a, b). Wt a-syn labeling is clearly visible for cells transfected with low amounts (5 μg) of plasmid, corresponding to low expression levels (Supplementary Fig. 1c), and substantially brighter for those transfected with high amounts (25 μg) of plasmid, corresponding to high expression levels (Supplementary Fig. 1d). These images also demonstrate that >90% of all cells expressing VAMP8-pHluorin also express co-transfected a-syn (Supplementary Fig. 1c, d).

We employed western blots to estimate the absolute amounts of Wt a-syn expressed in RBL cells after transfection with the lower and higher amounts of DNA, which we calibrated using purified recombinant a-syn (Fig. 2d and Supplementary Fig. 2). Converting to cellular concentrations, we determined the low expression level to be 130 μg/ml or 9 μM and the high expression level to be 300 μg/ml or 20 μM. These values fall within the range reported for concentrations of a-syn found in living neurons (5–50 μM or 75–750 μg/ml).^24^

We quantified differences in expression levels of Wt a-syn and several a-syn mutants, A30P, A53T, and G51D, using flow cytometry (as gated by VAMP8-pHluorin expressing cells). These measurements show a mean fluorescence 3–4-fold higher for cells transfected with the higher concentration of plasmid (25 μg) compared to those transfected with the lower concentration (5 μg) (Fig. 2e and Supplementary Fig. 1e). For experiments described in subsequent sections, we used fluorescence imaging and standard reference samples to ensure the level of expression for a-syn variants was consistent among samples transfected and tested on different days (see Materials and methods section).

*A-syn variants at low expression levels differentially inhibit exocytosis stimulated by both antigen and thapsigargin*. We used confocal microscopy to quantify exocytosis as the normalized increase in VAMP8-pHluorin fluorescence (Supplementary Movies 2a, b; Supplementary Fig. 3a–d) for RBL cells co-transfected with a-syn and stimulated with either antigen or thapsigargin. This confocal imaging approach measures net exocytosis without accounting for any endocytosis that would result in reacidification and consequent fluorescence quenching. However, we used bafilomycin, a proton pump inhibitor, to determine that little or no endocytosis and reacidification of vesicles occurs on the timescale of our confocal measurements (Supplementary Fig. 4). As described in a later section, we also analyzed the TIRF movies (Fig. 2a–c, Supplementary Movies 1a–c) in which we counted individual exocytotic events directly and obtained consistent results.

We initially stimulated cells with a low dose of antigen and found that Wt a-syn, as well as PD-linked a-syn mutants A53T and E46K, cause strong inhibition (70–85%) compared to pcDNA controls (Supplementary Fig. 3c–g). By comparing VAMP8-phluorin with VAMP7-pHluorin (a marker for secretory granule/lysosomes^25,26^) these experiments also showed that the significant inhibition by a-syn we observe is selective for stimulated exocytosis of REs (Supplementary Fig. 3h). We tested whether inhibition of exocytosis by a-syn is discernable with high-dose antigen stimulation and determined that it is not: stimulating co-transfected RBL cells first with a low dose of antigen shows inhibition, but this is abrogated if followed by a much higher dose, which elicits exocytosis levels comparable to those in cells with the vector control (Supplementary Fig. 3i, j). These results show that higher antigen doses overcome the inhibitory mechanism detected with low-dose antigen and also that the a-syn-mediated inhibition we observe is not due to cytotoxicity.

**Fig. 3:**
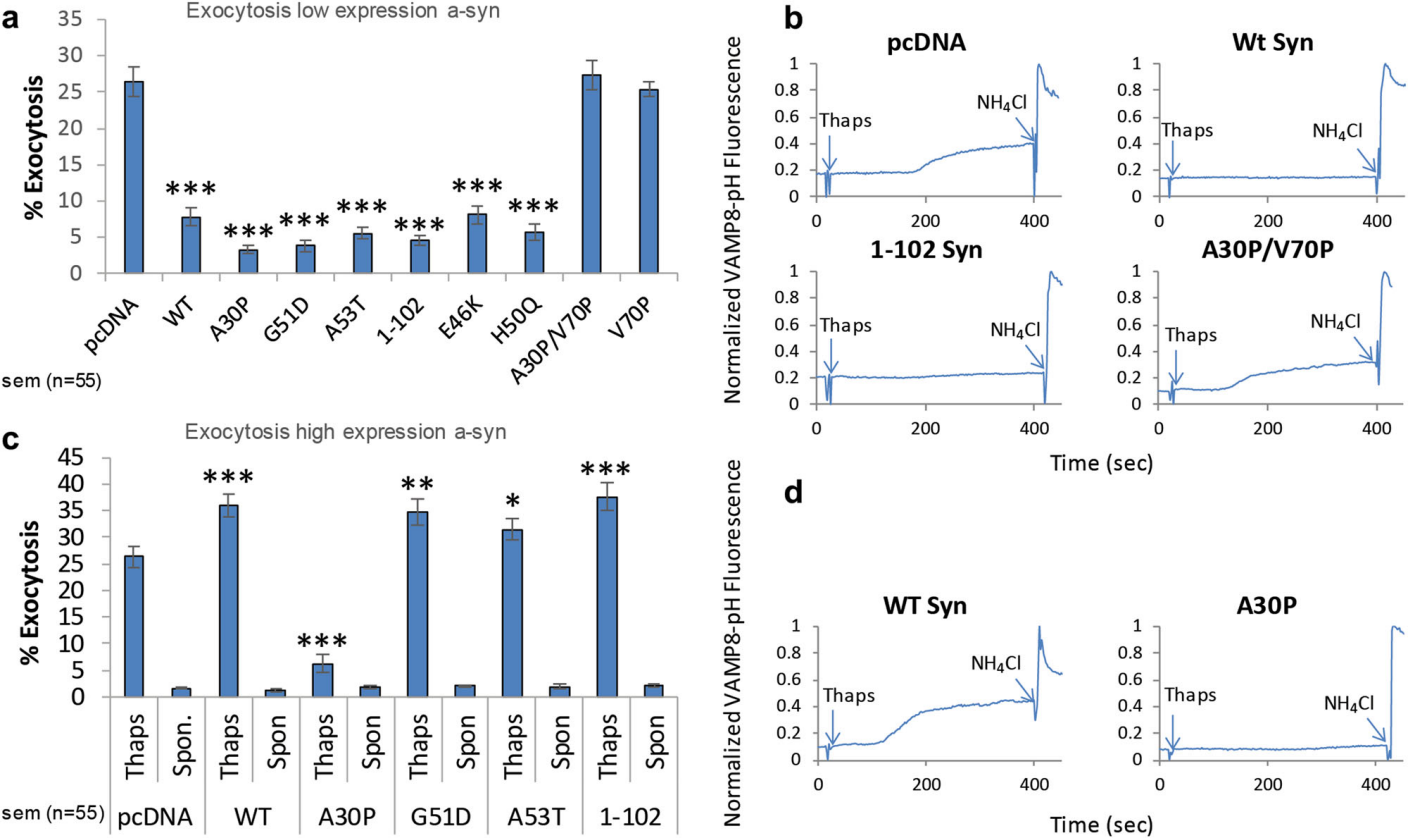
Low expression levels of Wt a-syn inhibit stimulated exocytosis of REs, whereas high expression levels cause enhancement, and mutants of a-syn show distinct differences. As indicated, RBL cells were co-transfected with VAMP8-pHluorin and low (5 μg **a**, **b**) or high (25 μg **c**, **d**) levels of pcDNA, Wt a-syn or a-syn mutants. VAMP8-pHluorin fluorescence increase was monitored in confocal movies (similar to Supplementary Movies 2a, b) before and after addition of thapsigargin (250 nM) to stimulate exocytosis, and after addition of NH_4_Cl (50 mM, 300–400 s after stimulation) to dequench intracellular VAMP8-pHluorin fluorescence. **a** Averaged exocytosis stimulated by thapsigargin in cells with low a-syn expression levels (*n* = 55); spontaneous release for each of these samples is less than 2.5% as shown in Supplementary Fig. 5. **b** Representative traces of VAMP8-pHluorin fluorescence integrated from movies for **a** of multiple confocal fields of 5–6 cells (all traces shown in Supplementary Fig. 5). **c** Averaged exocytosis stimulated by thapsigargin in cells with high a-syn expression levels (*n* = 55). **d** Representative traces of VAMP8-pHluorin fluorescence integrated from movies for **c** as in **b** (All traces shown in Supplementary Fig. 9). All data sets shown are from 3 to 4 independent experiments; Error bars are ±SEM; ***P-values < 0.001, **P-values < 0.01, *P-values < 0.05

*Low level expression of Wt a-syn, PD-linked and C-terminal mutants, but not V70P mutants, inhibit exocytosis*. Using thapsigargin as a stimulant, we quantified inhibitory effects of Wt and a broad range of a-syn mutants with confocal microscopy. At low expression levels, Wt a-syn and PD-linked mutants G51D, A53T, E46K, and H50Q all inhibit thapsigargin-stimulated exocytosis of REs (Fig. 3a and Supplementary Fig. 5a–d), by 70–85% compared to the vector control, similar to that observed for antigen stimulation (Supplementary Fig. 3).

We tested the generality of our results using PC12 cells, which are derived from the adrenal medulla, secrete dopamine, and have been used as a model system for neurosecretion.^22^ Transfected PC12 cells expressing VAMP8-pHluorin and pcDNA show 25% exocytosis stimulated by thapsigargin (Supplementary Fig. 6a). This stimulated response is inhibited by 70% for cells transfected with Wt a-syn expressed at low concentrations (5 μg) (Supplementary Fig. 6b, c), consistent with our results with RBL cells (Fig. 3a, b).

Previous studies reported that the disordered C-terminus of a-syn (Fig. 1) mediates interactions with proteins involved in exocytosis, including the v-SNARE VAMP2^27^ and Rab family proteins,^28^ which also participate in stimulated exocytosis of REs in RBL cells.^23^ Evaluating cells transfected at low levels (5 μg), we found that truncated a-syn 1–102 (C-terminal residues 103–140 deleted) inhibits thapsigargin-mediated exocytosis similarly to Wt a-syn (Fig. 3a, b). We also tested Wt a-syn tagged with mRFP on its C-terminus (Wt syn-mRFP), considering that a fluorescent tag the size of mRFP (~27 kDa) may sterically disrupt some cellular interactions of a-syn (~14 kDa), and found it to be similarly inhibitory (Supplementary Fig. 7a, b, e). These results indicate that the C-terminus is not involved in the inhibition of stimulated exocytosis we observe at low expression levels of Wt a-syn.

We evaluated the contributions of selected residues within the N-terminal amphipathic region of a-syn (residues 1–100, Fig. 1), which has been shown to be necessary for membrane binding.^29^ The PD-linked mutant A30P perturbs the first of two alpha helical regions formed in this region^30,31^ and decreases membrane affinity.^32^ A30P a-syn at low expression levels inhibits stimulated exocytosis under our conditions (Fig. 3a). To further reduce a-syn membrane binding, we introduced a valine to proline mutation at residue 70 within the A30P mutant (A30P/V70P). The V70P mutation is located in the second helical region of membrane-bound a-syn (Fig. 1) and was previously shown to decrease membrane binding when combined with an alanine to proline mutant at position 11 (A11P/V70P).^29,33^ Interestingly, A30P/V70P a-syn mutant does not inhibit thapsigargin-stimulated exocytosis under the same conditions (Fig. 3a, b), indicating that some aspect of membrane binding is essential for the inhibition we observe. We then examined the single V70P mutation and observed that it too fails to inhibit thapsigargin-stimulated exocytosis (Fig. 3a), indicating that perturbation in the helix-2 region of membrane-bound a-syn is sufficient and the A30P mutation is not required to disrupt this inhibitory effect.

**Fig. 4:**
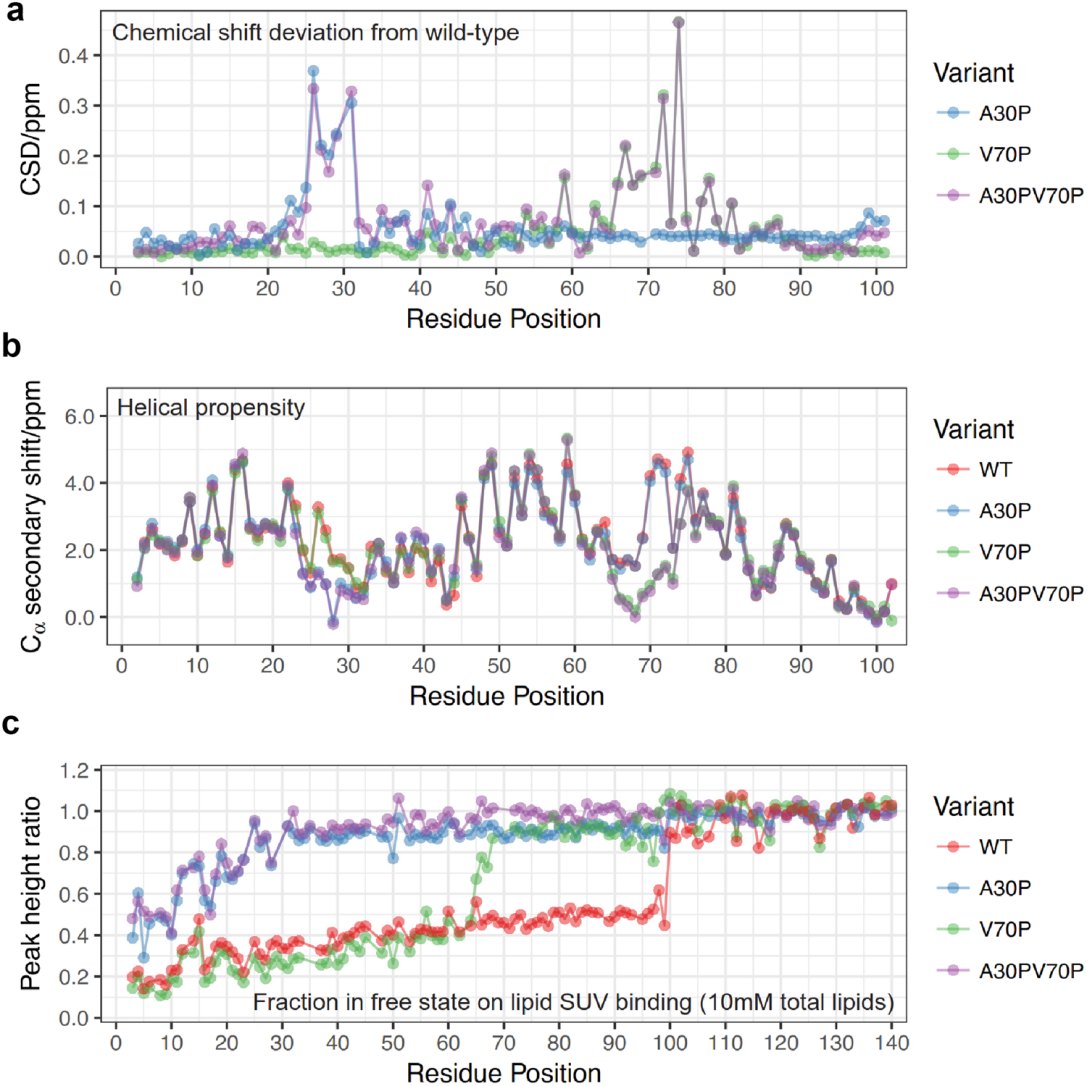
The V70P mutation perturbs helical micelle-bound structure locally; A30P and V70P mutations cause release from vesicle membranes of a-syn regions C-terminal to the mutation sites. **a** Average amide chemical shift deviation (CSD) of different a-syn mutants used in the study from Wt a-syn, all in the micelle-bound state, calculated as described in Methods section. Only the N-terminal 102 residues are plotted as the C-terminal region does not interact with the micelle. **b** C_α_ secondary chemical shift of a-syn variants in the micelle-bound state, calculated for each residue as the difference between the observed chemical shift and the tabulated chemical shift in a random coil conformation. Positive values indicate helical structure. **c** Vesicle binding of full-length a-syn variants measured as the ratio of NMR resonance intensities in the presence and absence of liposomes. The peak intensity ratio, representing the free fraction of each residue, is plotted for 50 μM protein with small unilamellar vesicles (SUVs) containing 10 mM total phospholipids at a molar ratio of DOPC:DOPE:DOPS = 60:25:15

*V70P mutation perturbs membrane binding affinity of a-syn helix-2, but not of helix-1, whereas A30P disrupts binding to vesicles*. To assess the structural consequences of the A30P and V70P mutations, alone or in combination, for membrane-bound a-syn we carried out NMR measurements on the micelle-bound and lipid vesicle-bound forms of the protein. The vesicle-bound state of a-syn is expected to closely resemble the state of the protein when bound to undocked SVs in vivo.^16,34,35^ The micelle-bound state of the protein has no direct physiological analog, but we and others have postulated that this state resembles the structure of a-syn when it is bound simultaneously to two different membrane surfaces, for example in a mode bridging the vesicle and plasma membranes, or potentially bridging two adjacent SVs.^16,17,36^ Analysis of ^1^H/^15^N chemical shift changes compared to Wt a-syn indicates that the V70P mutation alters the environment of sites as far away as 15 residues from the site of the mutation in the micelle-bound state (green trace, Fig. 4a). Deviations of Cα shifts from random coil values, which are indicative of secondary structure (green trace, Fig. 4b), indicate that helix-1 is unperturbed in the V70P mutant, whereas helix-2 exhibits reduced helicity around the site of the mutation, with a severe reduction of helical structure spanning about 10 residues centered on the site of the mutation. Together, these changes indicate that the V70P mutation does not perturb membrane binding associated with helix-1, but does locally perturb and deform the membrane-bound conformation of helix-2.

The vesicle-bound state of a-syn cannot be directly visualized using solution state NMR because of the slow tumbling time of lipid vesicles. Instead, NMR allows for observation of any parts of the protein that are not bound to the vesicle surface (and are therefore highly mobile). For any given signal originating from a specific residue site in the protein, the intensity of the signal, normalized by the intensity of the same signal in the absence of lipids, indicates the fraction of the protein population for which that protein residue is not bound to the vesicle surface. For Wt a-syn, these intensity ratio plots demonstrate that the entire membrane-binding domain of the protein, consisting of residues ~1–100, bind to the vesicle surface largely as a single unit (red traces, Fig. 4c and Supplementary Fig. 8). Indeed, more detailed structural studies have indicated that in this binding mode, helix-1 and helix-2 fuse into a single extended helix (Fig. 1b).^37–40^ For the V70P mutant, binding to the membrane surface is dramatically reduced for locations C-terminal to the site of the mutation, whereas binding for locations N-terminal to the mutation site is largely unaffected, and, in fact, appear to be slightly enhanced (green traces, Fig. 4c and Supplementary Fig. 8). This indicates that the proline at position 70 leads to detachment from the membrane surface of the portion of the protein following this mutation site, but not of the preceding portion.

We compared the effects on micelle and vesicle binding of the V70P mutation to those of the A30P mutation (blue traces) and of the A30P/V70P double mutant (purple traces) (Fig. 4 and Supplementary Fig. 8). As previously reported,^30,31^ the A30P mutation locally perturbs the structure of helix-1 in the micelle-bound state (blue traces, Fig. 4a, b), and leads to decreased vesicle binding of a-syn residues both N-terminal to and C-terminal to the site of mutation (blue traces, Fig. 4c, Supplementary Fig. 8), consistent with the reduced membrane affinity of this mutant. The effects of the double A30P/V70P mutation appear to be decoupled and additive in the micelle-bound state, with both helix-1 and helix-2 exhibiting local perturbation of helical structure, but with the micelle-bound structure remaining otherwise intact (purple traces, Fig. 4a, b). In contrast, in the vesicle-binding assay, the effects appear to be cumulative, with a dramatic loss of binding around the A30P site, and an additional small decrease in binding occurring C-terminal to the V70P site (purple traces, Fig. 4c and Supplementary Fig. 8). Our collective results show that the effects of the A30P mutation dominate the binding of a-syn to isolated vesicles, yet this mutant inhibits stimulated exocytosis similarly to Wt, while V70P does not. Thus, it appears that the disruption of helix-2 structure by V70P in the micelle-bound state, which may reduce its capacity to bridge between different membranes or possibly perturb another functional interaction, underlies the loss of its capacity to inhibit stimulated exocytosis.

*High expression of Wt a-syn enhances stimulated exocytosis of REs*. Cells transfected with the higher concentration of plasmid (25 μg) and expressing the higher levels of Wt, A53T, G51D, and 1–102 human a-syn show robust thapsigargin-stimulated exocytosis (Figs. 2a, c, 3c, d and Supplementary Fig. 9). Further, these responses show consistent enhancement over the thapsigargin-stimulated exocytic response observed with transfection of pcDNA alone (Fig. 3c). Exocytosis data for cells transfected at both low and high expression levels of a-syn variants were typically collected on the same day. We used pcDNA transfected at low levels as a positive control, consistently exhibiting stimulated exocytosis values of about 25%. Expression at high levels of Wt, A53T, G51D, and 1–102 a-syn increase this value by 20–40% (Fig. 3c). pcDNA transfected at the higher concentrations gave somewhat variable results but consistently showed stimulated exocytosis similar to or less than that with the lower concentration of pcDNA. We found that Wt a-syn-mRFP also loses its capacity to inhibit exocytosis when expressed at higher levels and shows a 20% increase in stimulated exocytosis when compared to mRFP alone expressed at high levels (Supplementary Fig. 7c–e). These results confirm expression level-dependent interactions of a-syn: for multiple constructs, low expression levels cause inhibition, whereas high expression levels cause enhancement, of thapsigargin-stimulated exocytosis of REs.

In contrast to Wt a-syn and other mutants tested at high expression levels, A30P a-syn does not exhibit loss of inhibition as compared in the same experiment (Fig. 3c, d and Supplementary Fig. 9). In a separate experiment we found that V70P a-syn, which does not inhibit exocytosis at low expression levels (Fig. 3a), also slightly enhances exocytosis at high expression levels (data not shown). Thus, it appears that while the interaction mode of a-syn with isolated vesicles is not critical for the capacity of a-syn to inhibit exocytosis, this interaction mode, and/or the structure of helix-1, both of which are perturbed by the A30P but not by the V70P mutation, may be important for stimulating vesicle exocytosis at higher expression levels.

**Fig. 5:**
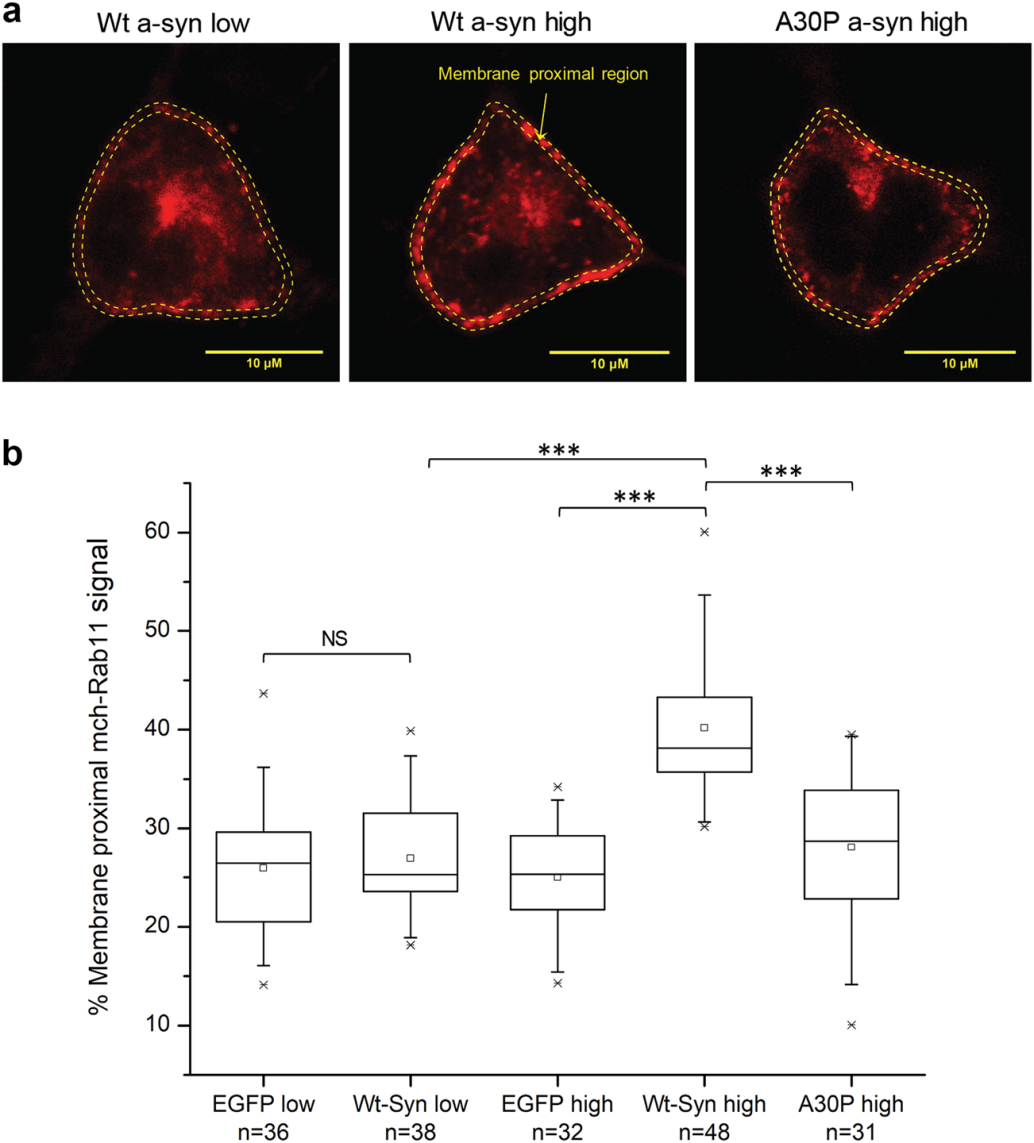
High expression levels of Wt, but not A30P, a-syn shift the distribution of REs toward the plasma membrane. RBL cells were co-transfected with mCh-Rab11 to label REs, and low or high levels of EGFP or Wt a-syn, or high levels of A30P a-syn. **a** Confocal micrographs of mCh-Rab11 fluorescence (REs) co-transfected with low levels (5 μg) of Wt a-syn or high levels (25 μg) of Wt or A30P a-syn. The fluorescence intensity within a thin shell around the plasma membrane (dashed lines) divided by total cell fluorescence was used to calculate % membrane proximal REs; scale bar = 10 μm. **b** Averaged % membrane proximal REs for specified samples in a box plot; number of cells evaluated for each condition is specified. The box represents 25th–75th percentile of the data, the midline represents the median and the small square represents the average. ***P-values < 0.001; NS, not statistically significant (*P*-values > 0.05)

*High expression levels of Wt a-syn cause dispersal of REs from the endocytic recycling compartment (ERC)*. To investigate possible explanations for differential effects on stimulated exocytosis at different a-syn expression levels we transfected cells with Wt a-syn or EGFP (as a control) together with mCh-Rab11, which labels REs.^23^ As shown in Fig. 5 we observed markedly differential effects on the distribution of REs: Higher, but not lower, expression levels of Wt a-syn cause redistribution of the REs away from the perinuclear region of the cell, corresponding to the ERC, and toward the plasma membrane. Quantifying this redistribution as the ratio of membrane proximal to total Rab11 fluorescence, the value for Wt a-syn at high expression levels is 40% compared to 27% for Wt a-syn at low expression levels or 25% for EGFP at high expression levels, both highly significant differences (Fig. 5b). Moreover, the value for A30P a-syn at high expression levels is similar to that for Wt a-syn at low expression levels. These differential effects on RE distribution are thus parallel to those observed for stimulated RE exocytosis, particularly our observation that high levels of Wt a-syn but not A30P a-syn cause enhancement. It appears that proximity of abundant REs to the plasma membrane may underlie the enhancing effect.

We also used transfected VAMP8-phluorin to evaluate the redistribution of REs at different expression levels of Wt a-syn, using the same confocal movies from which we evaluated levels of exocytosis (similar to Supplementary Fig. 3a, b and Supplementary Movies 2a, b). Following stimulation with thapsigargin and subsequent addition of NH_4_Cl to deacidify all REs and thereby expose all VAMP8-pHluorin to neutral pH, we quantified the ratio of membrane proximal VAMP8-pHluorin fluorescence to total VAMP8-pHluorin fluorescence. As shown in Supplementary Fig. 10a, and similar to our quantification of Rab11 in Fig. 5, the ratio of membrane proximal fluorescence to total cellular fluorescence is significantly higher for cells expressing the higher level of Wt a-syn compared to cells expressing the lower level of Wt a-syn and compared to the cells transfected with the empty vector.

Supplementary Fig. 10b shows a second type of analysis of these same movies (similar to Supplementary Movies 2a, b), which yields further information about possible inhibition at the stage of exocytosis, i.e., the fraction of membrane proximal REs that are released after stimulation. In this case, we quantified the membrane proximal VAMP8-pHluorin fluorescence of stimulated cells before addition of NH_4_Cl as a measure of exocytosed REs, and after subsequent addition of NH_4_Cl as a measure of both exocytosed REs and membrane proximal REs that had not been exocytosed. The ratio of these two values provides a measure of the fraction of membrane-proximal REs released. Interestingly, we found that this ratio is significantly less for cells expressing high levels of Wt a-syn, compared to the control cells (Supplementary Fig. 10b), indicating inhibition at the stage of exocytosis despite increased amount of membrane proximal REs (Supplementary Fig. 10a). The cells expressing low levels of Wt a-syn exhibit the lowest fluorescence ratio, as expected from the inhibition we clearly observed for this condition in our other assays (Figs. 2b, 3a, b).

We also analyzed our TIRF movies (Fig. 2a–c; Supplementary Movies 1a–c) by counting individual exocytotic events as a function of time. As shown in Supplementary Fig. 11, the total number exocytotic events stimulated by thapsigargin is greater for cells expressing high levels of Wt a-syn compared to control cells transfected with the pcDNA empty vector (consistent with Fig. 3c), but the rate of release is somewhat lower, consistent with Supplementary Fig. 10b and pointing to a limiting factor in this case. The rate and overall extent of stimulated exocytotic events in cells expressing low levels of Wt a-syn are both significantly lower than in control cells. Together, these results are consistent with the view that the net stimulated exocytosis is enhanced for cells expressing high levels of Wt a-syn because of an increased number of membrane proximal vesicles (Fig. 5, Supplementary Fig. 10a) but that an inhibitory mechanism remains in place because the fraction of membrane proximal vesicles that can be exocytosed is reduced compared to the control (Supplementary Fig. 10b).

**Fig. 6:**
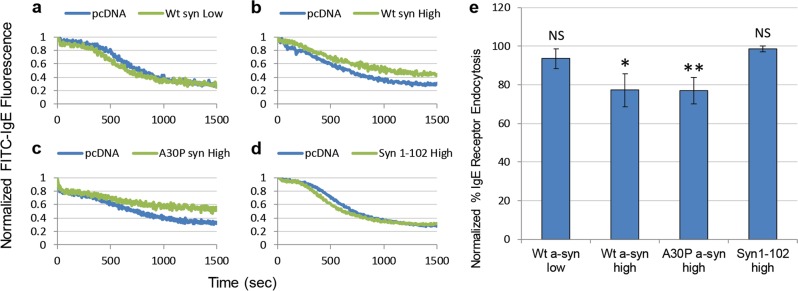
Higher expression levels of Wt a-syn inhibit stimulated endocytosis, depending on the C-terminal tail. RBL cells were co-transfected with mRFP, to mark positively transfected cells, and low levels (5 μg) of pcDNA or Wt a-syn **a**, or high levels (25 μg) of pcDNA and Wt a-syn **b**, A30P a-syn **c**, or 1–102 a-syn **d**. Cells were sensitized with FITC-IgE, incubated at 37°, and endocytosis was triggered with anti-IgE antibody. Endocytosis of FITC-IgE was monitored in a flow cytometer by FITC fluorescence quenching. Fluorescence values prior to stimulation were set to 1.0, and fractional fluorescence after stimulation were compared directly to the pcDNA sample run in parallel, corresponding to maximal endocytosis. **e** Endocytosis by indicated test samples were normalized to their paired pcDNA control samples (set at 100% endocytosis, not shown). Error bars are ± SD from three independent experiments. **P-values < 0.01, *P-values < 0.05; NS, not statistically significant (*P*-values > 0.05)

*Endocytosis stimulated by crosslinking IgE-receptors on RBL cells is inhibited at higher expression levels of Wt a-syn, depending on its C-terminal tail*. Several studies implicating a-syn as an important regulator of synaptic vesicle trafficking have focused on endocytic events,^41–44^ and we evaluated effects of a-syn on endocytosis in RBL cells stimulated by an external agent. Cells were transfected with mRFP as a reporter for positively transfected cells, together with either pcDNA or plasmids of a-syn constructs transfected at high (25 μg) or low (5 μg) levels. Cells were then labeled with IgE conjugated to fluorescein isothiocyanate (FITC-IgE), which displays bright fluorescence at the plasma membrane upon binding to its receptor, FcεRI. Crosslinking of FITC-IgE/FcεRI stimulates their endocytosis, and FITC fluorescence is quenched as it moves from the neutral pH environment of the extracellular space to the more acidic environment of the endosomal compartments.^45^ Cells transfected with pcDNA and stimulated with an anti-IgE antibody exhibit fluorescence quenching due to endocytosis as monitored by flow cytometry, and the a-syn constructs we tested in direct comparison in each experiment show some differences (Fig. 6). We found that Wt a-syn expressed at low levels causes little or no change in stimulated FITC-IgE endocytosis (Fig. 6a, e). In contrast, Wt a-syn expressed at high levels causes a modest but significant inhibition (Fig. 6b, e). Unlike the case with stimulated exocytosis (Fig. 3c, d) A30P expressed at high levels inhibits stimulated endocytosis similarly to Wt a-syn (Fig. 6c, e). Interestingly, the truncated 1-102 mutant does not inhibit stimulated endocytosis (Fig. 6d, e), suggesting that a functional C-terminus is involved in this activity. Consistently, we found that Wt a-syn-mRFP, which is C-terminally tagged, also fails to inhibit stimulated endocytosis (data not shown).

**Fig. 7:**
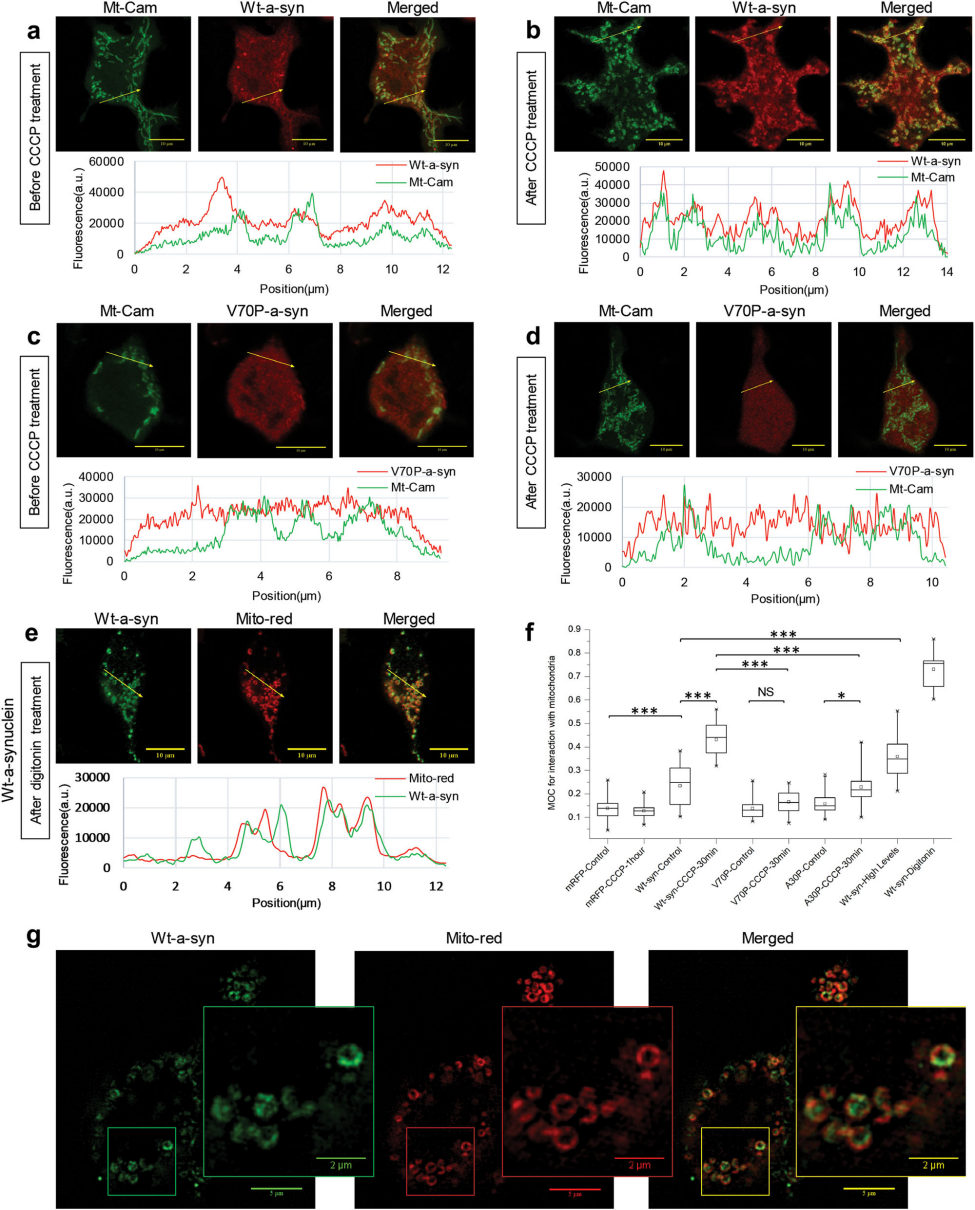
Mitochondrial and cellular stress cause association of Wt a-syn with mitochondria, disrupted by perturbation of helices 1 and 2. RBL cells co-expressing low levels of Wt a-syn **a**, **b** or V70P a-syn **c**, **d** together with Mito-cameleon (green) were incubated for 30 min at 37^o^ in BSS without **a**, **c** or with **b**, **d** 10 µM CCCP then fixed, and a-syn was immunostained (red) for visualization with confocal microscopy; scale bar = 10 μm. Alternatively, RBL cells expressing Wt a-syn were labeled with MitoTracker Red, washed with cold PBS and permeabilized with 0.001% digitonin for 3 min **e**. Cells were then fixed, and a-syn was immunostained (green) and visualized as for other samples. Traces below micrographs in **a**–**e** are fluorescence intensities across the arrow-line drawn on the images for both a-syn and mitochondrial marker channels. **f** Averaged Mander’s overlap coefficients (MOC) for a-syn and mitochondrial marker were calculated for specified samples as represented in a box plot. The box shows 25th–75th percentile of the data; the midline shows the median, and the small square shows the average. ***P-values < 0.001, NS, not statistically significant (*P*-values > 0.05). **g** Samples prepared as described in **e** were visualized at super resolution with structure illumination microscopy, using fiduciary beads in each sample to ensure the channel alignment; scale bar is 5 μm for the image and 2 μm for the inset

*A-Syn at high expression levels binds to internal membranes depending on formation of N-terminal helices*. We observed that cells expressing higher levels of a-syn exhibit intracellular puncta that can be visualized with immunostaining. Because a-syn is known to aggregate within neurons, we looked, but found no evidence, for a-syn aggregates in Western blots of our transfected cells (e.g., Supplementary Figure 2). We tested several different conditions in these experiments, including using the chemical crosslinker disuccinimidyl glutarate to capture metastable oligomers,^46^ cell-sorting for a-syn transfected cells to increase signal-to-noise, and immunoprecipitation of a-syn to reduce background, prior to western blotting (data not shown). Overexpressed a-syn has been reported to co-localize with various internal organelle membranes including lipid droplets.^47^ We found that higher expression levels increase co-localization of Wt a-syn with the lipid droplet marker Nile Red in RBL cells (Supplementary Fig. 12a, b). Co-localization with lipid droplets is greatly reduced for the A30P mutant, even at high expression levels (Supplementary Fig. 12c), suggesting that it is driven by membrane-binding of the N-terminal region of a-syn. We also observed increased co-localization of Wt a-syn with the mitochondrial marker Mito-chameleon (Mt-cam) at higher expression levels (Fig. 7f, Supplementary Fig. 13), as has been previously reported for HEK cells with a high degree of a-syn over-expression^48^ and with neurons under some conditions.^49^

*A-syn co-localization with mitochondria increases after mitochondrial stress and involves helices 1 and 2*. We quantified the association of transfected a-syn constructs with matrix-labeled mitochondria using both simple line scans (Fig. 7a–e) and more comprehensive cross-correlation (Fig. 7f). The latter approach allows a rigorous statistical analysis, and we evaluated differences using transfected mRFP as a nonspecific, baseline reference. Wt a-syn at low expression binds detectably to mitochondria, and this colocalization increases at the high expression level (Fig. 7a, f). To investigate conditions that may enhance co-localization, we considered that PD-linked genes constitute the PINK1/Parkin pathway for mitophagy, which is triggered by mitochondrial stress.^50^ We tested two different conditions expected to cause mitochondrial stress: collapse of the mitochondrial membrane potential by the ionophore CCCP, and mild permeabilization of cells by low concentrations of digitonin which releases cytoplasmic ATP (as well as non-bound a-syn). We found that both of these stress conditions lead to a dramatic co-localization of a-syn with mitochondria (Fig. 7b, e, f). Higher resolution, provided by structured illumination microscopy reveals that a-syn is present in regions of stressed mitochondria adjacent to, but distinct from the mitochondrial matrix (Fig. 7g).^51^

We found that, compared to WT, both a-syn mutants V70P (Fig. 7c, d) and A30P exhibit reduced mitochondrial localization in the absence of mitochondrial stress and a reduced increase in co-localization under conditions of CCCP stress (Fig. 7f). Together with effects of A30P and V70P mutations on membrane interactions measured with NMR (Fig. 4), these results indicate that intact helix-1 and helix-2 structures are involved in a-syn association with mitochondria, which increases markedly under stress conditions.

## Discussion

**Fig. 8:**
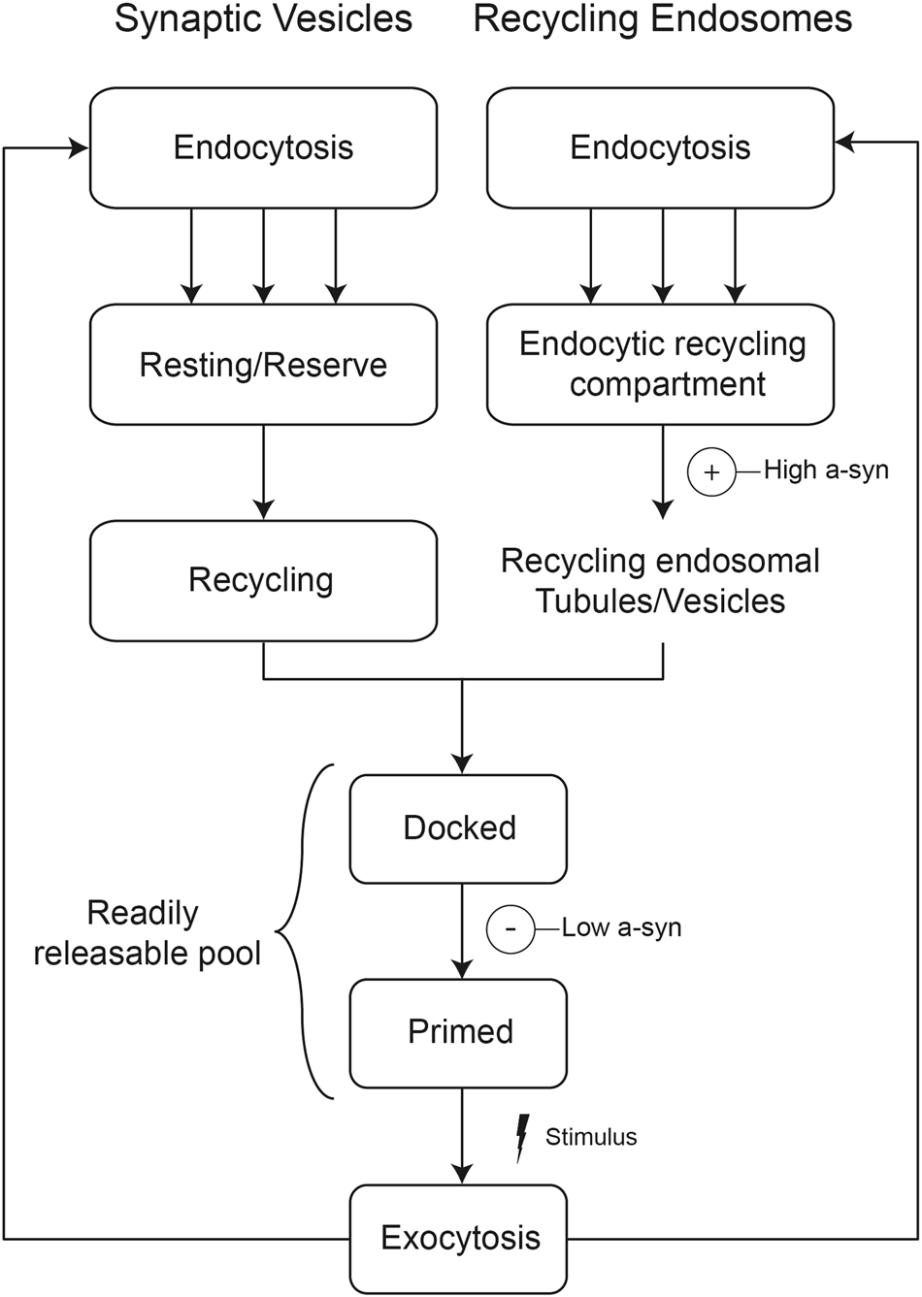
REs and SVs exhibit parallels in trafficking pathways and may be similarly affected by a-syn. Both SVs and REs are endocytosed by multiple processes and move through several endosomal vesicle stages, which we suggest have parallel features. We find that Wt a-syn at high expression levels (indicated by “(**+**)–High a-syn” in the diagram) increases the number of REs dispersed from the ERC toward the plasma membrane (Fig. 5 and 10a), and suggest that this increased flux enhances the total level of stimulated exocytosis (Fig. 3c); the capacity of a-syn to bind isolated vesicles (such as we observe in NMR measurements, Fig. 4c) is required for these effects. At low expression levels, Wt a-syn inhibits stimulated exocytosis of both REs (Fig. 2a) and SVs^75^ (indicated by “(**−**)–Low a-syn” in the diagram), with the effect on REs depending on an intact helix-2 (Fig. 4a, b). We suggest a-syn stabilizes the endosomal vesicles (REs or SVs) via broken helix binding at the docked stage, inhibiting release and decreasing the total level of stimulated exocytosis

Intracellular trafficking of membrane vesicles is observed to be abnormal in many neurodegenerative disorders, including PD,^52^ and our findings with REs in model RBL cells are consistent with a growing body of literature showing that a-syn regulates stimulated, as well as homeostatic, trafficking of vesicles in a variety of cells, including of SVs in neurons.^41,53–56^ Figure 8 shows simplified trafficking parallels between SVs in neurons and REs in RBL cells, based on recent reviews.^57,58^ Endosomal trafficking to sort and recycle membrane components occurs in most cell types, including neurons in which it likely intersects with specialized SV trafficking networks.^57,59–61^

Both stimulated RE exocytosis in RBL cells and stimulated SV exocytosis in neurons rely upon an accessible pool of vesicles that is replenished by endocytosis. Because RBL cells contain very little (or no) endogenous a-syn (Supplementary Figs. 1 and 2), we could monitor effects of Wt, PD-linked and other selected mutants of human a-syn, acutely transfected into these cells. We evaluated structural features of this protein as related to their in vitro binding properties, their binding to intracellular structures, and their functional effects (Table 1). The phenotypes we have characterized suggest that a-syn’s structural interactions with membranes involved in trafficking processes are similar for REs and SVs.

We observed distinctive effects depending on whether the RBL cells expressed “low” vs. “high” levels of Wt a-syn. The corresponding cellular concentrations, as quantified by western blotting (Fig. 2d), are in the range reported for neurons (5–50 μM^24,62^). Comparison of fluorescent labeling intensity by flow cytometry showed our high expression level to be 3–4 times the concentration of our low expression level (Fig. 2e). Low expression levels of Wt a-syn inhibit exocytosis of REs, stimulated either by low-dose antigen crosslinking of IgE-receptors (Supplementary Fig. 3), or by thapsigargin, which circumvents the receptor by directly activating downstream signaling to increase cytoplasmic Ca^2+^ (Fig. 3a, b). Low expression levels of Wt a-syn also inhibit thapsigargin-stimulated exocytosis in PC12 cells, a dopamine releasing, rat cell line (Supplementary Fig. 6) that has been used as an experimental proxy for neuronal exocytosis and found previously to be inhibited by a-syn.^22^

Our analysis of a panel of a-syn mutants reveals that inhibition of stimulated exocytosis at low a-syn expression levels occurs with all PD-linked mutations tested (E46K, A53T, G51D, H50Q, A30P). Inhibition also occurs with C-terminal truncation (1–102), indicating that previously described SNARE engagement or other interactions mediated by this segment are not involved in this inhibitory property.^7^ However, inhibition is eliminated by substitution of a proline residue at position 70 (V70P or A30P/V70P) (Fig. 3a, b). This residue is located in the middle of the second of two separated helices that are formed by a-syn on the surface of detergent or lysophospholipid micelles (Fig. 1c). We have postulated that this broken-helix binds simultaneously to two juxtaposed membranes, engaging docked SVs via a-syn helix-1 while binding the adjacent plasma membrane via a-syn helix-2.^17,35,37,63,64^ This double-anchor interpretation is consistent with observations that a-syn binds more tightly to membrane-associated SVs than to isolated SVs.^65^ Although V70P significantly deforms the helix-2 structure in the region near the mutation site (Fig. 4a, b) it has little effect on the capacity of a-syn to bind to intact isolated vesicles (Fig. 4c and Supplementary Fig. 8). Therefore, we postulate that inhibition of stimulated RE exocytosis requires the interactions of intact helix-2 with the plasma membrane, which would allow specific engagement of docked vesicles via simultaneously binding of helix-1. A similar double-anchor interaction of Wt a-syn has been recently proposed by others^36^ and suggested as one of several possible mechanisms contributing to inhibition of SV exocytosis in neurons by over-expressed a-syn.^14^

The A30P mutation has been shown to reduce the affinity of a-syn for SVs^66^ consistent with our measurements with synthetic vesicles (Fig. 4c, Supplementary Fig. 8). Because this mutation alone does not mitigate inhibition of stimulated exocytosis in our assay (Fig. 3a), nor in previous reports of exocytosis inhibition in PC12 or adrenal medullary cells,^22^ we suggest that under these conditions helix 1(A30P) binding to vesicles remains strong enough to facilitate tight binding via the broken-helix bridge to the plasma membrane, as long as helix 2 is intact.

To relate RE trafficking more directly to neurons we consider the SV trafficking cycle (Fig. 8). As for REs, SV endocytosis replenishes pools necessary for exocytosis, and this inward trafficking process can occur through distinctive routes, some of which appear to intersect with endosomal pathways from which discrete SVs bud.^59–61^ Distinguishable SV pools have been variously described, and in Fig. 8 we refer to three major pools as “Resting/Reserve,” “Recycling”, and “Readily Releasable,” as described by Alabi and Tsien.^58^ Similar pools have been characterized in many other studies, in terms of their proximity to the “active zone” (AZ) of the synapse, SV clustering involving connectors or tethers, and level of stimulation required for pool activation.^14,58^ Previous studies report that human a-syn prevents neurotransmitter release, in part due to inhibition of SV recycling following exocytosis, thereby reducing the recycling and readily releasable pools.^41,56,67–70^ Our characterization of a-syn inhibition by broken-helix binding to docked vesicles suggests additional explanations for those previous observations, involving direct effects that impede post-docking processes such as SV priming and/or fusion.

Our experimental system further reveals that high expression levels of Wt a-syn cause an overall increase in the level of stimulated exocytosis (Fig. 2c, h). Our western blot analysis and microscopic visualization provide no evidence of stable a-syn aggregates in the cytoplasm that might remove an inhibitory species. While we cannot conclusively exclude the presence of less stable oligomeric a-syn species or aggregated species associated with internal membranes, a number of observations are inconsistent with the notion that the formation of such species could explain the effects we observe at high expression levels of a-syn. First, since these cells do not contain detectable quantities of endogenous a-syn, it is difficult to rationalize how sequestration of monomeric a-syn in oligomers at high expression levels could enhance exocytosis beyond the levels observed for controls (Fig. 3c). Second, the A30P mutation is a potent driver of a-syn oligomer formation,^71^ yet this mutant does not restore or enhance exocytosis at high expression levels (Fig. 3c, d). Third, high expression levels of a-syn lead to a redistribution of Rab11 positive recycling endosomes compared to both low levels of a-syn and to controls, and this effect would be difficult to explain by recruitment of monomeric a-syn into oligomers (Fig. 5). Finally, our analyses of VAMP8 fluorescence before and after NH_4_Cl (Supplementary Fig. 10b) and individual exocytic events in our TIRF data (Supplementary Fig. 11) suggest strongly that a-syn retains a measure of inhibitory function even at high expression levels, again arguing against an aggregation-mediated loss of inhibition.

Our results therefore point to a separate function of a-syn that may involve other aspects of trafficking and resulting availability of vesicles for exocytosis. We found that enhancement of stimulated RE exocytosis at higher Wt a-syn expression levels is unaffected by C-terminal truncation and all but one PD-linked mutations tested. However, the A30P mutation does abrogate this effect (Fig. 3c, d), indicating that the structural requirements for this faciliatory function of a-syn differ from those of its inhibitory function. There have been previous reports suggesting a-syn enhancement of SV exocytosis,^72^ including one study in which a-syn was found to enhance the kinetics of individual vesicle release events by altering the dynamics of fusion pore formation/closure.^73^ To interpret our results for stimulated outward trafficking and release of REs tin RBL cells, we considered that a-syn binding to membranes is curvature dependent, with stronger binding to more highly curved membranes.^74–76^ A-syn is thought to stabilize high membrane curvature^14^ and can even tubulate membranes,^77,78^ raising the possibility that a-syn plays a role in dispersing vesicles generated via tubulation of larger membrane structures such as the ERC (Fig. 8). Consistent with this, we observe a reduction in the perinuclear ERC and an increase in plasma membrane proximal vesicles in the presence of high levels of a-syn (Fig. 5 and Supplementary Fig. 10a). We also found that a high antigen dose (200 ng/ml DNP-BSA) overcomes the inhibition by low expression a-syn (Supplementary Fig. 3i, j), possibly by generating more recycling vesicles from the perinuclear ERC.^79^ We suggest that a-syn’s capacity for stabilizing membrane curvature underlies the enhancement of RE exocytosis we observe at high expression levels, changing trafficking distributions and yielding a greater availability of vesicles for docking at the plasma membrane. Consistent with this, we found that the A30P mutation, which reduces affinity of a-syn for vesicles, fails to enhance exocytosis (Fig. 3c, d) or to increase membrane-proximal vesicles at high expression levels (Fig. 5). This reduced affinity appears to be insufficient for stabilizing the curvature of endocytic tubules and dispersing vesicles from the ERC via the low affinity extended-helix state.

A recent study found that SVs in the distal/reserve pool are more tightly clustered in neurons from α/β/γ-synuclein knock-out mice compared to Wt mice,^80^ with the total SV pool size remaining constant. Consistent observations were made by Nemani et al.^68^ who showed in mouse brain slice neurons and in transfected neurons from the hippocampus that over-expression of a-syn disrupts SV clustering while also slightly shifting the distribution of vesicles distally from the AZ. The total SV pool was again found to be similar for over-expressed a-syn as for Wt. Our results with REs in RBL cells are in many ways consistent with those of Vargas et al.^80^ and Nemani et al.^68^ In our model, high levels of Wt a-syn promote vesicle release from the ERC (Fig. 8), thereby increasing the pools of REs that are readily available for exocytosis at the plasma membrane. We expect that inhibitory interactions detected at low expression levels of a-syn (Fig. 3a) also occur at high expression levels of a-syn as suggested by our experiments showing a reduced release rate of membrane proximal vesicles (Supplementary Figs. 10b, 11). However, we surmise that net exocytosis is enhanced because many more vesicles are available, consistent with the view that the facilitating interactions of a-syn predominate over those that provide negative regulation. We postulate that Nemani et al.^68^ observed only inhibition with over-expressed a-syn and not enhancement because synaptic terminals are very small relative to RBL cell surfaces, and SV clusters are necessarily located in close proximity to AZs. Thus a-syn driven dispersion of SV clusters appears to shift the population further from the AZ, effectively reducing the recycling and readily releasable pool. In fact, Nemani et al.^68^ offered a somewhat similar explanation for their observations that the A30P mutation in a-syn abrogates inhibition of SV release in neurons but does inhibit release of chromaffin granules in adrenal medullary cells:^22^ the round shape of the latter cells (more similar to RBL cells) does not require a-syn targeting to a spatially restricted release site within axonal synaptic terminals.

Importantly, although the effect of a-syn to disperse clustered vesicles may yield different consequences for vesicle exocytosis in different cellular contexts, our results strongly indicate that this dispersive effect is a general function of a-syn that is distinct from its role in directly modulating fusion of docked vesicles, and furthermore suggest a structural basis for these different functions. Interestingly, clustering of SVs was recently proposed to be mediated by liquid–liquid phase separation of the presynaptic protein syanpsin.^81^ It is possible that the ERC, which appears as a collection of tubulated and vesiculated membranes, may also form via this recently discovered mechanism for intracellular organization. This suggests the further intriguing possibility that a-syn’s capacity to disperse SV clusters and to disperse REs from the ERC results from a-syn facilitating vesicle release from such phase-separation-induced structures.

The endocytosis of IgE/FcεRI stimulated by anti-IgE that we investigated in RBL cells differs somewhat from that occurring with SVs in neurons and REs in RBL cells, which both correspond to compensatory inward trafficking to recycle the exocytosed vesicles.^19^ However, key features such as curvature and fission of the invaginating membranes are likely to be similar, and a-syn may contribute to or interfere with these processes. Our observation that low expression levels of a-syn have no significant impact on stimulated endocytosis, whereas high expression levels are weakly inhibitory and depend on the C-terminal tail (Fig. 6), further demonstrates that the function of a-syn is variable with concentration and structural interactions in RBL cells. Our observations that high expression levels of Wt a-syn enhance stimulated RE exocytosis but have only a modest capacity to inhibit endocytosis suggest that the facilitating effect of increasing the pool of accessible vesicles by enhancing dispersal from the ERC is dominant.

Our search for aggregated a-syn in RBL cytoplasm at high expression levels led us to observe, instead, the association of a-syn with lipid droplets and mitochondria (Fig. 7, Supplementary Figs. 12 and 13), as has been reported previously.^47,48,72,82–85^ Co-localization of a-syn with mitochondria is particularly intriguing as a-syn has been reported to interfere with mitochondrial fission/fusion, transport, and autophagy in neuronal systems, and disruption of these functional dynamics is associated with PD.^49^ Indeed, the helical state of vesicle-bound a-syn is reminiscent of long amphipathic helices in mitofusions that facilitate mitochondrial membrane fusion.^86^ A-syn’s capacities for interacting with other proteins, broken-helix binding, and curvature sensing could also affect fission/fusion and thereby other activities.

We also examined the effects of mitochondrial and cellular stress, in part because the PINK1/Parkin mitochondrial stress response pathway is strongly implicated in PD.^87^ Both treatment with the ionophore CCCP and mild permeabilization of the plasma membrane by digitonin substantially increases co-localization of low expression a-syn with mitochondria, and co-localized a-syn is distinct from the mitochondrial matrix as visualized with super-resolution imaging (Fig. 7g). Both a-syn mutations, A30P and V70P, cause significantly lower co-localization, with and without stress treatments (Fig. 7f), suggesting that membrane binding by both helix-1 and helix-2 is involved. Interestingly, a-syn has been reported to interact with the mitochondrial import machinery.^88^ During import, the inner and outer mitochondrial membranes are brought into close proximity,^89^ potentially presenting a-syn with a high affinity binding site consisting of closely apposed membranes, and requiring an intact helix-2 structure. Furthermore, the membrane composition of the mitochondrial inner membrane is rich in the negatively charged lipid cardiolipin, which may help to recruit a-syn.^90^ A-syn interactions with the mitochondrial import machinery may allow the normally cytosolic protein to cross the mitochondrial outer membrane, or a-syn may transport into mitochondria via a recently discovered mitochondrial proteostatic mechanism.^91^ Another possibility is that a-syn associates with mitochondria at mitochondrial-associated membranes, as has been observed previously,^92^ via broken-helix binding and an intact helix-2. Comparing the effects of the A30P and V70P mutations on mitochondrial co-localization to their effects on a-syn-mediated modulation of stimulated RE exocytosis suggests that a-syn first engages mitochondrial outer membranes via an extended helix (Fig. 1b) and then more tightly via broken-helix binding to a juxtaposed membrane (Fig. 1c).

In conclusion, we used RBL cells as a model system to study functional interactions of human a-syn, a protein with a strong link to PD but with a poorly understood function in neurological processes. These experiments allowed us to identify a regulatory role in stimulated exocytosis and endocytosis of endosomal vesicles, processes thought to be highly susceptible to neurodegeneration. We found that human a-syn can both inhibit and enhance stimulated exocytosis, depending on the expression levels of the protein. We further observed that a-syn co-localizes with mitochondria, increasing with mitochondrial and cellular stress. Intrinsic properties of a-syn binding to membranes appear to play a key role in most or all of these functions and localizations. Future experiments will aim to extend our results more directly to neurons, with the goal to clarify further the different types of membrane interactions involved in physiological function and how these are related to dysfunctional interactions that result in pathology.

## Methods

### Cell culture

RBL-2H3 cells were cultured as monolayers in minimal essential medium (Invitrogen Corp, Carlsbad, CA) with 20% fetal bovine serum (Atlanta Biologicals, Atlanta, GA) and 10 µg/ml gentamicin sulfate (Invitrogen) as previously described.^93^ PC-12 cells were cultured in Dulbecco’s modified eagle medium (Invitrogen) with 10% fetal bovine serum and 10 µg/ml gentamicin. Adherent cells were harvested by treatment with Trypsin-EDTA (0.05%) for 8–10 min (RBL-2H3 cells) or 2–3 min (PC-12 cells), 3–5 days after passage. The RBL-2H3 cells are continuously cultured in the Baird-Holowka laboratory, routinely checked for normal functions, and frozen stocks are thawed for fresh cultures as warranted. Similarly, the PC12 were obtained from the laboratory of George Hess (Cornell University) where they were previously cultured and characterized.

### Reagents

Thapsigargin and phorbol 12-myristate-13-acetate were purchased from Sigma-Aldrich (St. Louis, MO). Trypsin-EDTA, 0.2 µm TetraSpeck™ beads, Alexa Fluor 488-labeled, Alexa Fluor 568-labeled, and Alexa Fluor 647-labeled goat anti-mouse IgG secondary antibodies were acquired from Invitrogen (CAT#: A21121, A21124, A21240; 1:200 dilution). Mouse monoclonal IgG_1_ anti-α-synuclein antibodies 3H2897(CAT#: sc-69977; 1:200 dilution) and 42/α-Synuclein (CAT#: 610787; 1:200 dilution) were purchased from Santa Cruz Biotechnology (Dallas, TX) and BD Biosciences (Franklin Lakes, NJ), respectively.

### Cell expression plasmids

cDNA for cell expression of human Wt a-syn, A53T a-syn, and E46K a-syn in pcDNA 3.0 vectors were obtained as a gift from Dr. Chris Rochet (Purdue). All other plasmids for cell expression of human a-syn mutants (A30P, E46K, G51D, V70P, A30P/V70P, Syn-1-102) were created within this vector by site directed mutagenesis using Phusion High-Fidelity DNA Polymerase (New England Biolabs). Plasmids for VAMP8-pHluorin and VAMP7-pHluorin were created as previously described.^23,94^ To create the Wt a-syn-mRFP plasmid, the cDNA encoding human Wt a-syn was introduced into a Clontech vector (Clontech) containing mRFP sequence, using Hind III and Kpn I restriction sites.

### Transfection by electroporation

RBL-2H3 and PC-12 cell lines were harvested 3–5 days after passage, and 5 × 10^6^ cells were suspended in 0.5 ml of cold electroporation buffer (137 mM NaCl, 2.7 mM KCl, 1 mM MgCl_2_, 1 mg/ml glucose, 20 mM HEPES, pH 7.4). Co-transfections used a reporter plasmid DNA (5 µg VAMP8-pHluorin or VAMP7-pHluorin, or 1.5 µg mRFP), together with 5 µg (“low” expression) or 25 µg (“high” expression) of human Wt a-syn (or control) plasmid DNA (pcDNA 3.0, Wt, A53T, E46K, A30P, G51D, H50Q, 1–102, A30P/V70P, or V70P). For antigen-stimulated exocytosis experiments, RBL cells were co-transfected with 12.5 µg of Wt, A53T, or E46K a-syn plasmid DNA. We found higher efficiency expression for plasmids in Clontech vectors, compared to pcDNA 3.0 vectors, and correspondingly we used 1.5 µg or 10 µg of mRFP and Wt-syn-mRFP a-syn plasmids for “low” or “high” expression experiments, respectively. We find for RBL cells that cells transfected with two constructs express both or none, such that a fluorescent construct can be used as a reporter for cells co-transfected with a non-fluorescent construct.

For all conditions cells were electroporated at 280 V and 950 μF using Gene Pulser X (Bio-Rad). Then cells were immediately resuspended in 6 ml medium and cultured for 24 h to recover; the medium was changed after live cells became adherent (1–3 h). For exocytosis experiments the cell suspensions were added to three different MatTek dishes (2 ml/dish) (MatTek Corporation, Ashland, MA) for recovery. For antigen-stimulated exocytosis experiments, cells were sensitized with 0.5 μg/ml anti-2,4-dinitrophenyl (DNP) IgE during the recovery period.^95^

We took several measures to ensure consistency, in multiple experiments over different days, of low or high expression levels of a-syn variants, corresponding to transfection with the 5 or 25 μg amounts of plasmid. As shown in Supplementary Fig. 1, we visualized the levels using immunofluorescence imaging (labeling with an antibody specific for all tested variants of a-syn) and quantified for several variants using flow cytometry. We also determined that RBL cells co-transfected with VAMP8-pHluorin and a-syn show a strong correlation with respect to fluorescence intensity from VAMP8-pHluorin compared to immunostained a-syn at each expression level. Therefore, in every experiment we evaluated VAMP8-pHluorin fluorescence as a reliable reference for consistency of transfection efficiency and thereby a measure of the consistency in transfection of a-syn at low (5 μg) and high (25 μg) levels. In addition, we regularly compared immunofluorescence with the a-syn-specific antibody to confirm that our VAMP8-pHluorin-based evaluation was consistently reliable.

### Stimulated exocytosis assays

After the electroporation recovery period and prior to imaging, cells were washed once and then incubated for 5 min at 37 °C with buffered saline solution (BSS: 135 mM NaCl, 5 mM KCl, 1 mM MgCl_2_, 1.8 mM CaCl_2_ 5.6 mM glucose, 20 mM HEPES, pH 7.4). For RBL cells VAMP8-pHluorin or VAMP7-pHluorin fluorescence was monitored for 20 s prior to addition of either 1 ng/ml DNP-BSA (antigen), or 250 nM thapsigargin, and after 6–8 min stimulation 50 mM NH_4_Cl was added. In one set of experiments, cells were first stimulated with 1 ng/ml DNP-BSA and then, after 6 min, stimulated with 200 ng/ml DNP-BSA. To evaluate possible contribution of endocytosis to measured exocytosis (Supplementary Fig. 4), cells were treated with bafilomycin (100 nM) just prior to stimulating with thapsigargin. PC-12 cells were stimulated with 100 nM phorbol 12-myristate-13-acetate at 20 s, and 250 nM thapsigargin at 5 min. Cells were monitored by confocal microscopy (Zeiss 710) using a heated, 40× water objective. VAMP8-pHluorin and VAMP7-pHluorin were excited using the 488-nm line of a krypton/argon laser and viewed with a 502–551 nm band-pass filter.

Offline image analysis was conducted using ImageJ (National Institutes of Health). Time traces of VAMP7-pHluorin or VAMP8-pHluorin fluorescence were normalized to a 0–1 scale using the following equation: (value(*t*) − minimum)/(maximum − minimum), with value(*t*) being the measured pHluorin fluorescence at a given time, minimum being the lowest monitored fluorescent value (basal, averaged prior to stimulation), and maximum being the averaged value following NH_4_Cl addition. The following equation was used:

1 $${\mathrm{\% }}\,{\mathrm{Exocytosis}} = \frac{{({\mathrm{Averaged}}\,{\mathrm{stimulated}}\,{\mathrm{fluorescence}} - {\mathrm{basal}}\,{\mathrm{fluorescence}})}}{{({\mathrm{Fluorescence}}\,{\mathrm{after}}\,{\mathrm{NH}}_4{\mathrm{Cl}} - {\mathrm{basal}}\,{\mathrm{fluorescence}})}} \times 100$$

For total internal reflection fluorescence (TIRF) microscopy, samples for exocytosis were prepared as described above, and single cells were imaged in TIRF mode with a Zeiss Elyra microscope. As for the exocytosis experiments monitored by confocal microscopy, cells were stimulated with 250 nM thapsigargin before addition of 50 mM NH_4_Cl at specified time points. Exocytosis events, appearing as spreading flashes of fluorescence, were quantified by manual counting (Supplementary Fig. 11).

### Immunostaining of a-syn variants

Cells were electroporated and incubated in MatTek dishes for the recovery period as described for particular experiments, then fixed with 4% paraformaldehyde +0.1% glutaraldehyde. Fixed cells were labeled in PBS with 10 mg/ml BSA using a monoclonal anti-a-syn antibody followed by an Alexa Fluor (488 or 568 or 647) conjugated secondary antibody, and then imaged by confocal microscopy or analyzed by flow cytometry.

### Distribution of recycling endosomes

Samples were electroporated as outlined above with 5 µg of mCherry-Rab11A plasmid DNA to label recycling endosomes^23^ and one of the following: 5 µg or 25 µg Wt a-syn, 25 µg A30P a-syn, or 3 µg or 15 µg EGFP plasmid DNA. Samples were fixed and immunostained with anti-a-syn antibody(3H2897) after the recovery period. Samples were confocally imaged using a 63× oil objective, selecting a plane near the middle of the cell including both plasma membrane and perinuclear regions. Fluorescent signal from a-syn immunostaining or EGFP was used to select the brighter cells at lower and higher expression levels, and Rab11A fluorescence was quantified to determine the relative distributions of REs using ImageJ (NIH) software. A shell of ~800 nm width was drawn to include the plasma membrane and a small region extending inward; the Rab11A fluorescence in that outer shell was divided by the total cellular fluorescence to yield the % REs proximal to the plasma membrane. A similar procedure was used to characterize distribution and exocytosis of membrane proximal REs when VAMP8-pHluorin was used as a marker, but in this case snapshots from movies of live cells (similar to Supplementary Movies 2a, b) were analyzed. In one type of analysis (Supplementary Fig. 10a) VAMP8-pHluorin fluorescence was measured after stimulation and subsequent addition of NH_4_Cl to reveal all exocytosed and intracellular REs; then the ratio of membrane proximal (outer shell) to total (whole cell) REs was determined (similar to Fig. 5). In a separate analysis to determine fraction of membrane proximal REs that are stimulated to exocytose (Supplementary Fig. 10b), the fluorescence in the outer shell at the end of the stimulation period was divided by the fluorescence in the same shell after subsequent addition of NH_4_Cl.

### Flow cytometry and immunofluorescence

After transfection by electroporation, samples were plated in 60 mm dishes for standard recovery period. Cells were then harvested, washed, and resuspended in BSS. To determine expression levels, cells were fixed and labeled with a-syn antibody (42/α-Synuclein). Samples were analyzed using a BD FACSAria Fusion Fluorescence Activated Cell Sorter, and data were quantified using FCS Express 5 Flow Research software. Analysis was gated to include single cells and positively transfected cells (VAMP8-pHluorin or mRFP). For quantification of Wt or mutant a-syn expression levels Alexa Fluor fluorescence was measured on VAMP8-pHluorin gated cells. For endocytosis experiments, cells were not fixed but treated as described below; mRFP expressing cells were gated, and quenching of FITC-IgE was monitored from that subset of cells.

### Western blots to determine a-syn concentration in cells

RBL-2H3 cells were co-transfected with 5 μg of mRFP and 5 or 25 μg of Wt a-syn plasmid DNA. After the standard recovery period cells were harvested, washed, and suspended in BSS, and sorted by flow cytometry at 37 °C. Approximately twenty percent of cells were identified to be transfected with Wt a-syn based on signal from the mRFP channel, and these were counted and collected for further analysis. Cells were washed in PBS, resuspended at 2 × 10^6^ cell equivalents/ml in lysis buffer (25 mM Tris, pH 7.4, 100 mM NaCl, 1 mM EDTA, 1% (v/v) Triton 100, 1 mM DTT, 1 mM sodium orthovanadate, 1 mM β-glycerol phosphate, 1 μg/ml leupeptin, and 1 μg/ml aprotinin), and supernatants were retained following microcentrifuge sedimentation. In some experiments the chemical crosslinker, disuccinimidyl glutarate, was added to stabilize possible a-syn oligomers as previously described.^46^ Standard solutions of recombinant Wt a-syn with concentrations of 2, 3.5, 5, 6.5, and 8 μg/ml were prepared by dilutions of a stock solution obtained by dissolving lyophilized, purified Wt a-syn in PBS buffer. After filtering through a 100 kDa cutoff filter, the concentration of the stock solution was determined by absorption at 280 nm using the calculated extinction coefficient of 5960 M^−1^ cm^−1^. The whole-cell lysates at 7.5 × 10^6^ cell equivalents/ml and standard Wt a-syn solutions were resolved by SDS/PAGE (25 μl/lane), and the proteins were transferred to PVDF membranes (Immobilon-P, Millipore). After fixation with 0.4% paraformaldehyde, the membranes were blocked in 10% BSA diluted in 20 mM Tris, 135 mM NaCl, and 0.02% Tween 20 and then incubated with anti-a-syn antibody (3H2897) diluted in the same buffer. Primary antibodies were detected with HRP-conjugated secondary antibodies followed by exposure to ECL reagent (Invitrogen).

After the blots were scanned, ImageJ was used to measure the density of each band, with the gel background subtracted. The density of bands corresponding to Wt a-syn protein in each cell lysate was normalized according to averaged density of three nonspecific bands in respective lane (loading control; <10% correction). Then the absolute of Wt a-syn protein in each cell lysate was interpolated from the calibration curve based on the densities of bands arising from the standard solutions of purified Wt a-syn. Amount of Wt a-syn per cell was calculated by dividing the amount of protein from each band by the known number of cell equivalents in the lane. Average volume of a cells was determined by transfecting RBL-2H3 cells with 5 μg of EGFP and confocal imaging z-stacks (1 μm thickness) of whole cell volume. Imaris image analysis software provided a 3D rendering of cells and determined the average volume of RBL-2H3 cell to be 2627 ± 983 μm^3^, based on measuring 50 cells.

### Mitochondrial stress assays

*CCCP treatment*. Samples were transfected with 5 μg of Wt a-syn plasmid DNA and 5 μg of Mito-chameleon. After recovery cells were washed twice with BSS at 37 °C, and incubated with 10 μM Carbonyl cyanide m-chlorophenyl hydrazone (CCCP) for 30 min. Samples were then fixed and immunostained with a-syn antibody (3H2897) and confocally imaged using a 63× oil objective.

Prior to mild digitonin permeabilization, samples were transfected with 5 μg of Wt a-syn, and after recovery, cells were washed twice with BSS buffer at 37 °C, stained with 200 nM MitoTracker® Red CMXRos at 37 °C for 30 min, followed by three washes with BSS buffer at 37 °C. Cells were then washed with cold PBS and permeabilized with 0.001% digitonin in cytosolic buffer (15 mM HEPES, 50 mM PIPES, pH 6.9, 1 mM MgSO_4_, 4 mM EGTA, 2 mM DTT, 1 μg/ml each of leupeptin, and aprotinin) for 3 min, then washed carefully with PBS. After fixing, samples were immunostained with a-syn antibody (3H2897) and confocally imaged using a 63× oil objective. For super resolution images of mitochondria within digitonin permeablized cells, structure illumination microscopy was used. Images were acquired on a Zeiss Elyra microscope utilizing a 63× oil objective, and 0.2 µM TetraSpeck™ beads were used as fiducial markers to ensure alignment of different imaging channels.

To quantify a-syn association with Mitochondria, 20–25 cells from three experiments for each condition (with and without stress) were analyzed as follows. The confocal image was split to a-syn and mitochondria fluorescence channels, and the fraction of signal from a-syn channel which overlaps with the signal from mitochondria was quantified as the Manders overlap coefficient using ImageJ via the JACoP plugin.

2 $${\mathrm{MOC}} = \frac{{\mathop {\sum }\nolimits_i \left( {R_i \times G_i} \right)}}{{\sqrt {\mathop {\sum }\nolimits_i R_i^2 \times \mathop {\sum }\nolimits_i G_i^2} }}$$

### Stimulated endocytosis assays

RBL cells were co-transfected with 5 μg of mRFP and 5 μg of pcDNA or 5 μg Wt a-syn plasmid DNA (low a-syn expression level) or 5 μg of mRFP and 25 μg of pcDNA or 25 μg of Wt a-syn, A30P a-syn, or 1–102 a-syn plasmid DNA (high a-syn expression level). After the recovery period, cells were harvested, washed and suspended in BSS, and incubated with 3 µg/ml FITC-IgE for 45 min at 37 °C. After washing, IgE-sensitized cells were analyzed by flow cytometry at 37 °C: IgE/FcεRI complexes were crosslinked by addition of anti-IgE antibody at *t* = 0 s. Acidification of internalized complexes was monitored by FITC fluorescence quenching, gated on cells positively expressing mRFP. Data were collected for ~1500 s, and analyzed with FCS Express 5 Flow Research software.

### Fluorescence detection of a-syn association with lipid droplets

Samples were transfected with 5 µg of Wt a-syn, 25 µg of Wt a-syn or 25 µg of A30P a-syn plasmid DNA, and after recovery washed twice with BSS at 37 °C. Cells were covered to protect from ambient light and incubated with 1 ml of Nile Red solution (1 ng/ml in 150 M NaCl) for 10 min at room temperature. After washing twice with 2 ml PBS and fixing, cells were immunostained with a-syn antibody (3H2897). Z-stack confocal images of whole cells were collected with a 63× oil objective. Fifty cells for each sample were imaged and quantified by counting the number of Nile Red positive lipid droplets labeled with Wt- or A30P a-syn over the whole cell volume.

### Statistical analyses for cell samples

Statistical analyses were performed with Prism software (Graphpad) and Microsoft Excel. Statistical significance was determined by a one-way ANOVA (Analysis of Variance) followed by Tukey’s post hoc test using Origin software. Level of significance is denoted as follows: *P < 0.05, **P *<* 0.01, ***P < 0.001.

### Expression and purification of recombinant isotope-labeled a-syn for NMR

Wt human a-syn cloned into a pT7-7 vector and a-syn mutants (A30P, V70P, A30P/V70P, and C-terminal truncation (1–102)) created using site-directed mutagenesis (Agilent QuikChange) were confirmed by Sanger sequencing. E. coli BL21 (DE3) cells were transformed with plasmid DNA and grown in M9 minimal media supplemented with ^15^N-ammonium chloride and ^13^C-glucose as the sole nitrogen and carbon sources, respectively. Recombinant protein production was induced by addition of IPTG at mid-log-phase growth period (OD_600_ = 0.6), and the cells were harvested 2–3 h after induction.

Cell pellets of full-length variants were lysed by sonication, followed by ultracentrifugation, acid precipitation of the resulting supernatant at pH 3.5 and precipitation by addition of 50% ammonium sulfate as previously described.^96^ The purified protein was lyophilized following dialysis into water. Truncation mutants are not acid-stable, and 1–102 a-syn was purified subsequent to cell lysis and ultracentrifugation using a series of ammonium sulfate cuts followed by anion exchange chromatography and reverse phase HPLC prior to lyophilization.^31,97^ Purity of the samples was assessed by SDS-PAGE, and subsequent NMR spectra provided additional confirmation of sample purity.

### NMR sample preparation

Lyophilized proteins were dissolved in NMR buffer (10 mM Na_2_HPO_4_, 100 mM NaCl, 10% D_2_O, pH 6.8) at an initial concentration of approximately 300 µM and filtered through a 100 kDa cutoff filter (Amicon Ultra 0.5 ml centrifugal, Millipore Sigma) to remove possible higher molecular weight oligomers. Protein concentration after filtration was assayed by absorbance at 280 nm using the calculated extinction coefficient of 5960 M^−1^ cm^−1^ for full-length variants. In addition to absorbance measurements, the integrated intensity of the amide proton region in the first plane of ^1^H-^15^N HSQC NMR spectra was used to generate samples of different a-syn variants at equal concentrations. Protein solutions were mixed with lipid or detergent stocks to generate vesicle-bound or micelle-bound samples.

### NMR of SDS micelle-bound a-syn

Samples of full-length or C-terminally truncated a-syn variants were mixed with deuterated (d25) SDS stock solution to a final SDS concentration of 40 mM. Triple resonance experiments (HNCA, HNCACB, CBCACONH) were conducted at 40 °C to determine peak assignments for resonances that moved relative to the assigned Wt a-syn spectrum and to measure the CA and CB chemical shifts.^98,99^ Data were collected on either a 600 or 700 MHz Bruker Avance spectrometers equipped with cryogenic probes and located at the Weill Cornell NMR Core facility or at the New York Structural Biology Center, respectively. Sensitivity-improved versions of the NMR experiments employing pulsed-field gradient were used.^100^ Typically for 3D experiments, 512 complex points were acquired in the ^1^H dimension centered on the water frequency (~4.7 ppm) with a spectral width of 10 ppm, 32 complex points were acquired in the ^15^N dimension centered at ~118 ppm with a spectral width of 26 ppm, and 64 complex points were acquired in the ^13^C dimension with the center frequency and spectral window optimized to increase resolution without aliasing the peaks. Spectra were processed using NMRPipe^101^ and visualization for sequential assignment was performed using CCPNmr Analysis.^98^ Spectra were referenced indirectly to DSS (2,2-dimethyl-2-silapentane-5-sulfonate) and ammonia using the temperature-adjusted chemical shift of water. For full-length V70P a-syn samples, the carbon dimension in triple-resonance spectra was re-referenced using the Wt a-syn protein by minimizing the average difference between the mutant and Wt chemical shifts for residues 111 to 130.

Amide group chemical shift deviations from Wt a-syn were calculated as

3 $$\Delta \delta _{{\mathrm{avg}}} = \sqrt {\frac{1}{2}\left( {\Delta \delta _{{\mathrm{HN}}}^2 + \frac{{\Delta \delta _{\mathrm{N}}^2}}{{25}}} \right)},$$

where $\Delta \delta _{{\mathrm{HN}}}$ is the amide proton chemical shift difference in ppm and $\Delta \delta _{\mathrm{N}}$ is the amide nitrogen chemical shift difference in ppm

Secondary carbon chemical shifts were calculated as

4 $$\Delta \delta _{\mathrm{C}} = \Delta \delta _{{\mathrm{C,measured}}} - \Delta \delta _{{\mathrm{C,random}}\,{\mathrm{coil}}},$$

where $\Delta \delta _{{\mathrm{C,measured}}}$ is the measured chemical shift, and $\Delta \delta _{{\mathrm{C,random}}\,{\mathrm{coil}}}$ is the sequence and temperature-corrected random coil chemical shift.^102,103^

### Lipid SUV vesicle binding assay

Lipid SUVs were prepared by sonication as described previously^99,104^ using a lipid mixture of DOPC:DOPE:DOPS at a molar ratio of 60:25:15, dried, hydrated using NMR buffer to a lipid concentration of 20 mM, sonicated until clarification, and ultracentrifuged to remove non-SUV membranes. This stock solution was mixed with a-syn protein stock solutions at defined ratios to generate samples for NMR spectroscopy at 5 and 10 mM total lipids. Matched lipid-free a-syn solutions were generated for comparison. Final protein concentrations were 50 µM for all samples in the SUV binding experiments. NMR samples were topped with argon gas, and ^1^H-^15^N HSQC spectra were acquired at 10 °C on the same day samples were prepared to preclude effects of lipid oxidation. Typically the spectra were acquired with 512 and 128 complex points, with spectral windows centered at ~4.7 and ~119 ppm, and with spectral widths of 14 and 26 ppm in the ^1^H and ^15^N dimensions, respectively.

Because the SUV-bound state tumbles slowly in solution, resonances from the bound protein are broadened beyond detection. Hence, any observed peaks represent protein residues not bound to the vesicle surface. The peak intensity ratio between lipid-containing and lipid-free samples is a measure of the fraction of the protein in which a given residue is unbound, and the intensity ratio profiles can be used to compare residue-per-residue SUV-binding for different a-syn variants.

## Supplementary information

Supplemental Material

Supplementary Movie 1a-TIRF-RBL-2H3-pcDNA-control-thapsigargin

Supplementary Movie 1b-TIRF-RBL-2H3-wt-syn-low-thapsigargin

Supplementary Movie 1c-TIRF-RBL-2H3-wt-syn-high-thapsigargin

Supplementary Movie 2a-Confocal-RBL-2H3-pcDNA-control-1ng-antigen

Supplementary Movie 2b-Confocal-RBL-2H3-wt-syn-low-1ng-antigen

## Data Availability

The western blots and fluorescence imaging, spectroscopy, and flow cytometry datasets generated during and/or analyzed during the current study are available from the corresponding authors on reasonable request. The NMR datasets generated during and/or analyzed during the current study are available from the corresponding authors on reasonable request.

## Supplementary information

**Supplementary information** accompanies the paper on the *npj Parkinson’s Disease* website (10.1038/s41531-019-0084-6).
