# Supplementary material for: Regulation of exocytosis and mitochondrial relocalization by Alpha-synuclein in a mammalian cell model
Source: NPJ Parkinsons Dis. 2019 Jun 27;5:12. doi: 10.1038/s41531-019-0084-6 (PMC6597712; doi:10.1038/s41531-019-0084-6)
Supplement: Supplementary file 1 — Supplemental Material [file 41531_2019_84_MOESM1_ESM.docx]

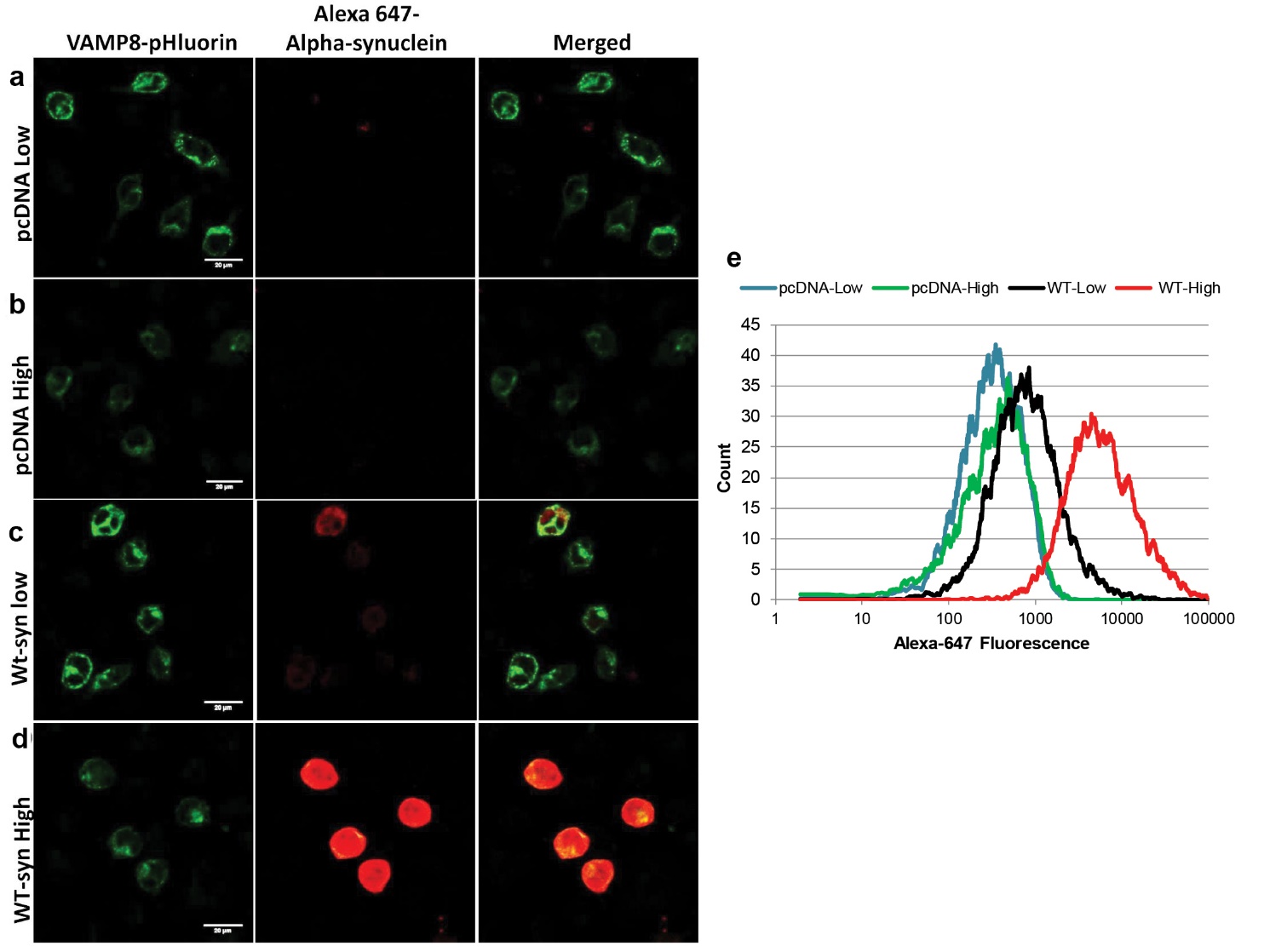


Supplementary Figure 1. **Low and high expression levels of a-syn are imaged and quantified using fluorescence microscopy and flow cytometry. a-d)** Representative confocal images of transfected RBL cells (scale bar = 20 µm). Cells co-expressing VAMP8-pHluorin and low levels of pcDNA (a) or Wt a-syn (c), or high levels of pcDNA (b) or Wt a-syn (d) were fixed, and a-syn was immunostained with Alexa-647. **e**) Cells prepared as described for (a-d) were analyzed using flow cytometry in which Alexa-647 fluorescence was measured in RBL cells gated by VAMP8-pHluorin fluorescence. A representative histogram of Alexa-647 fluorescence is shown with 5,000-6,000 VAMP8-pHluorin expressing cells analyzed for each condition.


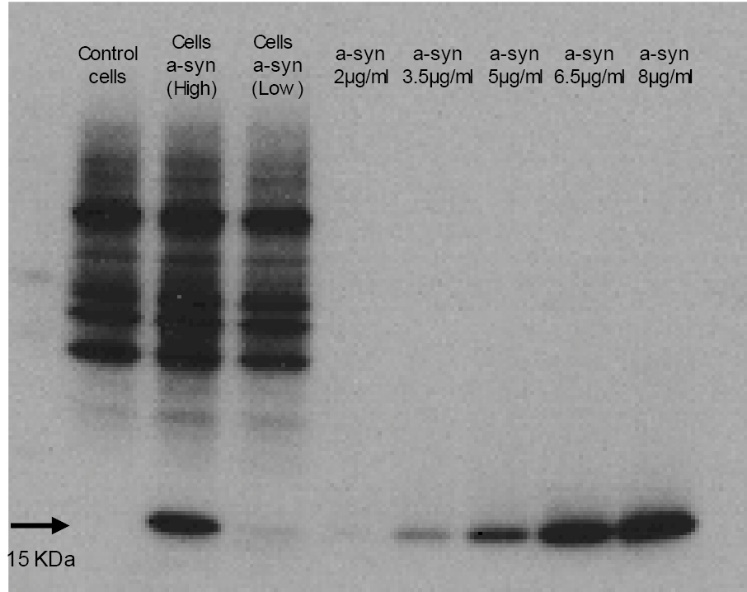


Supplementary Figure 2. **Concentration of Wt a-syn at low and high expression levels in RBL cells is estimated by western blotting.** Representative western blot from three separate transfections. Control RBL cells and cells transfected with low (5μg) and high (25μg) levels of Wt a-syn or pcDNA, co-transfected with mRFP for gating were counted and sorted by flow cytometer. Cell lysates containing 187,500 cells from each sample and standard solutions containing purified Wt a-syn at concentrations indicated were resolved by SDS/PAGE and detected by immunoblotting with anti-a-syn**.** Concentrations of a-syn expressed in cells, as derived from this blot and a calibration curve based on purified a-syn, are shown in Figure 2d. In this blot and those from other transfections, all samples were derived from the same experiment, processed in parallel, and run on the same gel.


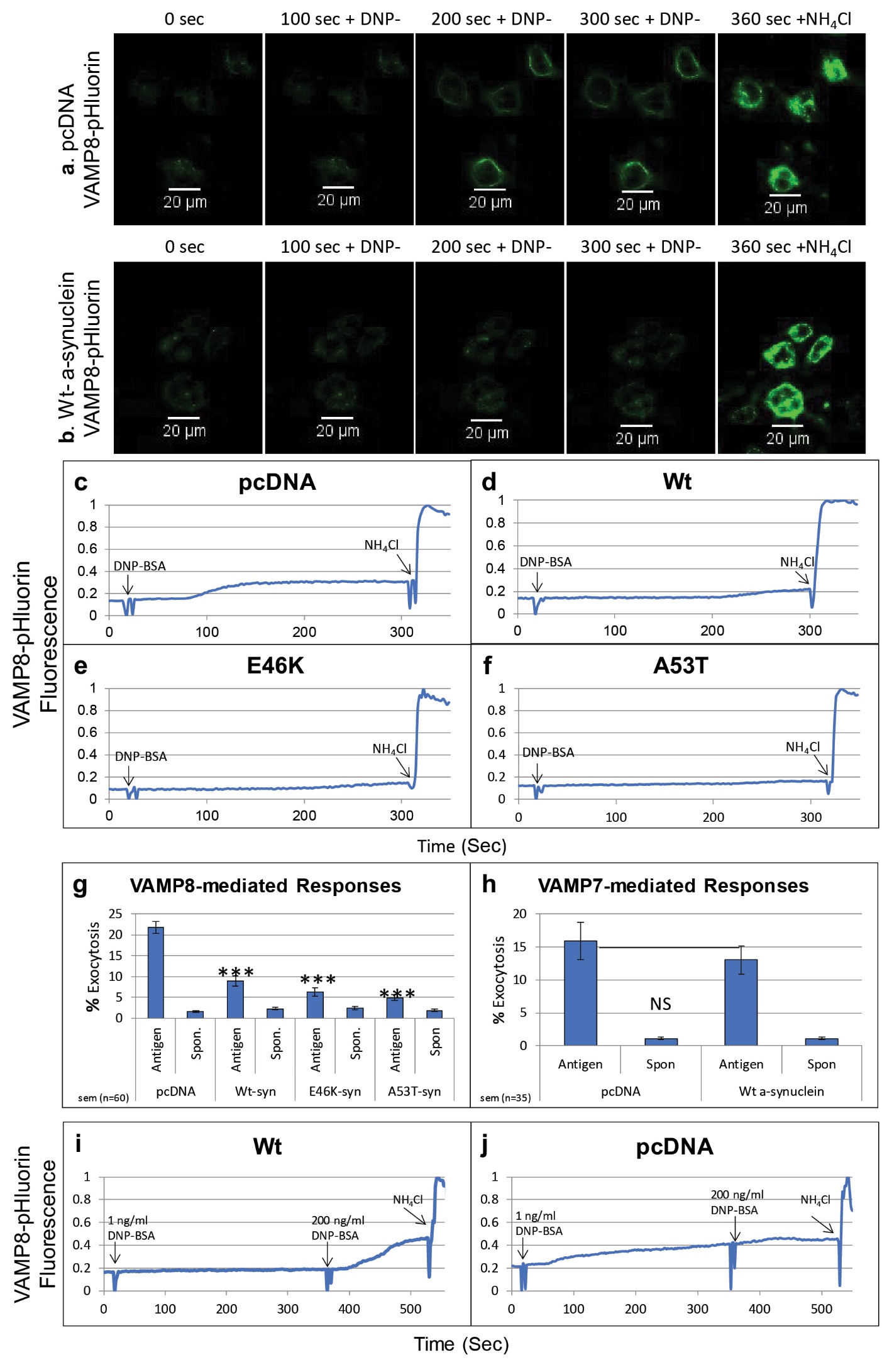


Supplementary Figure 3. **Exocytosis stimulated by antigen and effects of expressed a-syn variants and antigen dose are monitored with confocal movies.** RBL cells transfected with VAMP8-pHluorin and low levels of pcDNA (**a**) or Wt a-syn (**b**) were sensitized with anti-DNP IgE and stimulated with 1 ng/ml DNP-BSA at 20 sec; 50 mM NH_4_Cl was added at ~300 sec to neutralize the intracellular spaces and dequench all VAMP8-pHluorin fluorescence. Snapshots at times indicated are shown from Supplementary Movies 2a,b; scale bar = 20 μm **c-f**) Cells were co-transfected with VAMP8-pHluorin and pcDNA or Wt a-syn as in (a) and (b) or with low levels of E46K a-syn or A53T a-syn. Representative traces showing average change in VAMP8-pHluorin fluorescence following DNP-BSA addition are integrated from multiple fields of 5-6 cells in confocal movies, similar to Supplementary Movies 2a,b. **g and h**) Summary of three independent experiments monitoring fluorescence change in VAMP8-pHluorin (g: n=40 for each sample) or VAMP7-pHluorin (h: n=35 for each sample) in individual cells before (spon) or plateauing after DNP-BSA stimulation, normalized to fluorescence after addition of NH_4_Cl. Error bars are ± SEM; *** represents P-values <0.001, NS indicates values are not significantly different. **i-j)** RBL cells expressing VAMP8-pHluorin and Wt a-syn (i) or pcDNA (j) were sensitized with anti-DNP IgE and stimulated with 1 ng/ml DNP-BSA at 20 sec, and 200 ng/ml DNP-BSA at 360 sec. NH_4_Cl was added at 550 sec. Representative traces showing average change in VAMP8-pHluorin fluorescence were obtained as described above.


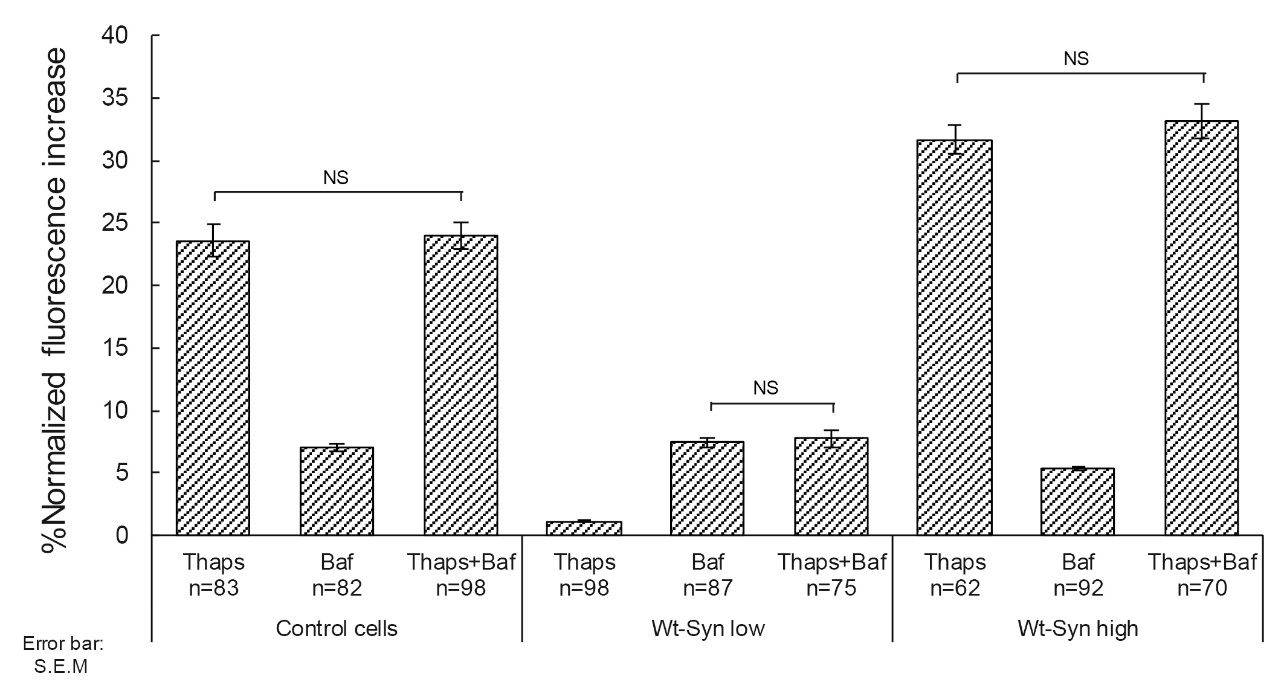


Supplementary Figure 4. **Bafilomycin experiment shows that RE endocytosis is slow on time scale of exocytosis.** RBL cells were co-transfected with VAMP8-pHluorin and, as labelled, pcDNA (control cells) or low (5μg) or high (25μg) levels of this plasmid containing Wt a-syn. Cells were treated (or not) with bafilomycin (100 nm) just prior to stimulating (or not) with thapsigargin (250 nM). As for Supplementary Figure 3, VAMP8-pHluorin fluorescence increase was monitored in confocal movies before and after stimulation, and after addition of NH_4_Cl (50mM, 300-400 sec after stimulation) to dequench intracellular VAMP8-pHluorin fluorescence. Fluorescence increases after the stimulation period were normalized by the fluorescence after addition of NH_4_Cl. Averaged change in normalized fluorescence is shown for each sample type and indicated number (n) of cells evaluated. All data sets shown are from 3 independent experiments; Error bars are ± SEM; NS = Not Significant, represents P-values >0.05. The small increase in VAMP8-pHluorin fluorescence observed for bafilomycin-treated cells during the same time period but that were not stimulated by thapsigargin may be due to slight deacidification of intracellular REs; this dequenched VAMP8-pHluorin fluorescence would not then increase further with exocytosis.


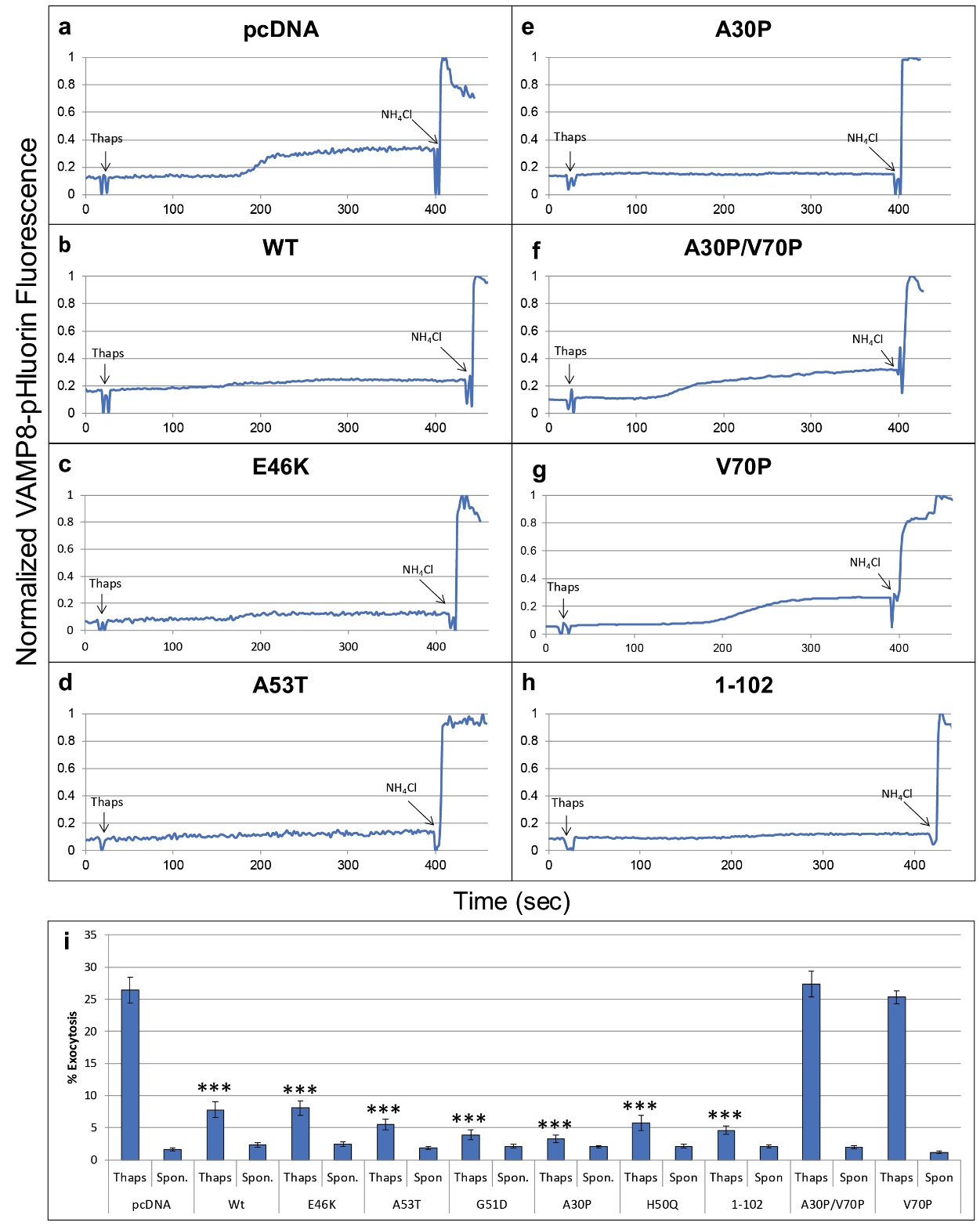


Supplementary Figure 5. **Wt a-syn** **and** **a-syn mutants except A30P/V70P and V70P expressed at low levels inhibit thapsigargin-stimulated exocytosis of REs.** RBL cells were co-transfected with VAMP8-pHluorin and low levels (5μg) of pcDNA (**a**) or Wt (**b**), E46K (**c**), A53T (**d**), A30P (**e**), A30P/V70P (**f**), V70P (**g**) or 1-102 (**h**) a-syn. All samples were stimulated with thapsigargin at t=20 sec, followed by addition of NH_4_Cl at t= ~400 sec. Representative traces showing average change in VAMP8-pHluorin fluorescence are integrated from multiple fields of 5-6 cells in confocal movies, similar to Supplementary Movies 2a,b. **i**) Summary of 3-4 independent experiments monitoring changes in VAMP8-pHluorin fluorescence in individual cells, before (spon) or plateauing after thapsigargin stimulation, normalized to fluorescence after addition of NH_4_Cl. Error bars are ± SEM for 55 individual cells for each condition; *** represents P-values <0.001.


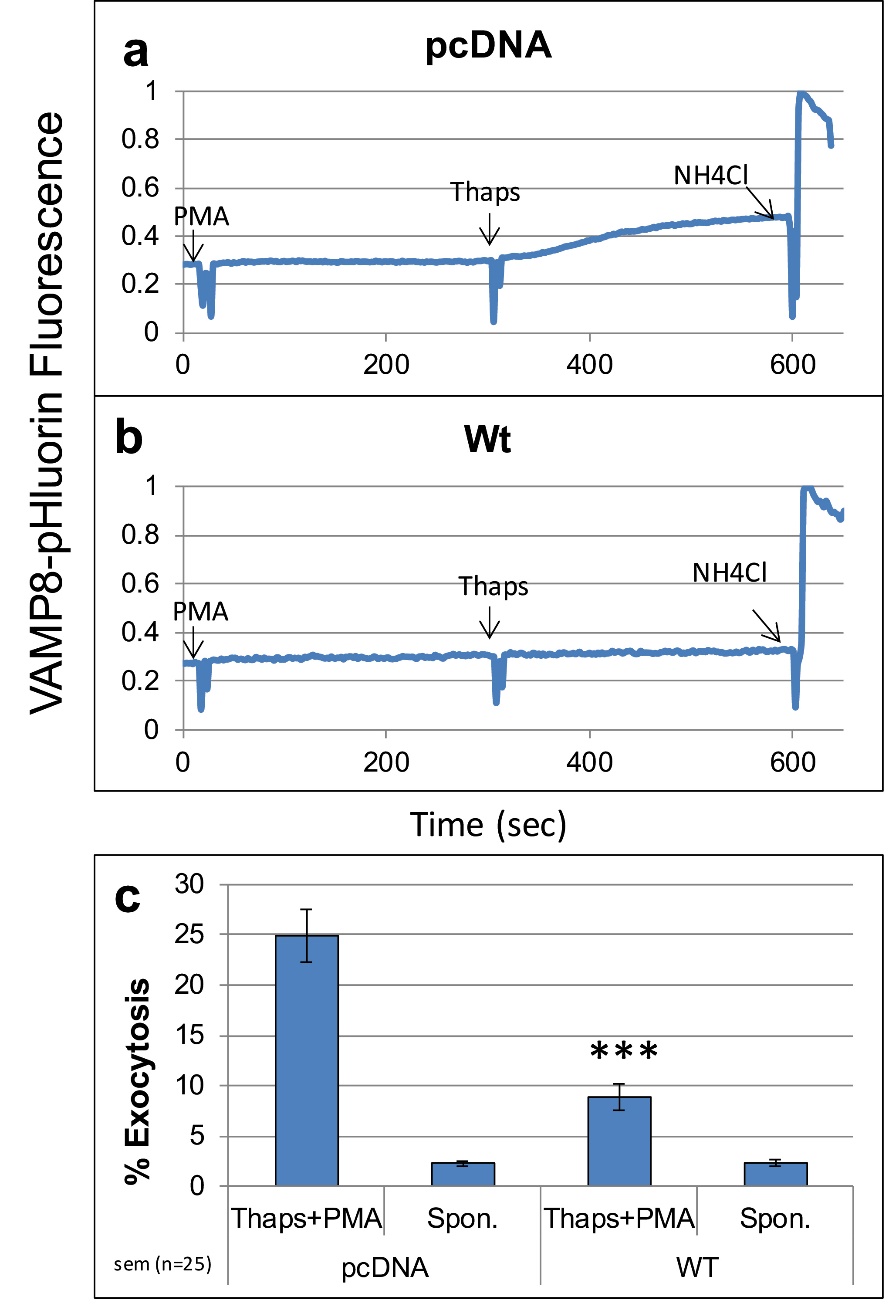


Supplementary Figure 6. **Low expression of Wt a-syn inhibits stimulated exocytosis in PC-12 cells.** Traces of stimulated exocytosis averaged from 5-6 individual PC-12 cells expressing VAMP8-pHluorin and low levels (5μg) of either pcDNA (**a**), or Wt a-syn (**b**). As indicated, phorbol 12-myristate-13-acetate (PMA) followed by 250 nM thapsigargin were added to stimulate exocytosis, and NH_4_Cl was added to dequench remaining intracellular VAMP8-pHluorin fluorescence. **c**) Averaged stimulated exocytosis determined from three independent experiments; error bars are ± SEM for 25 individual cells for each condition; *** represents P-values <0.001.


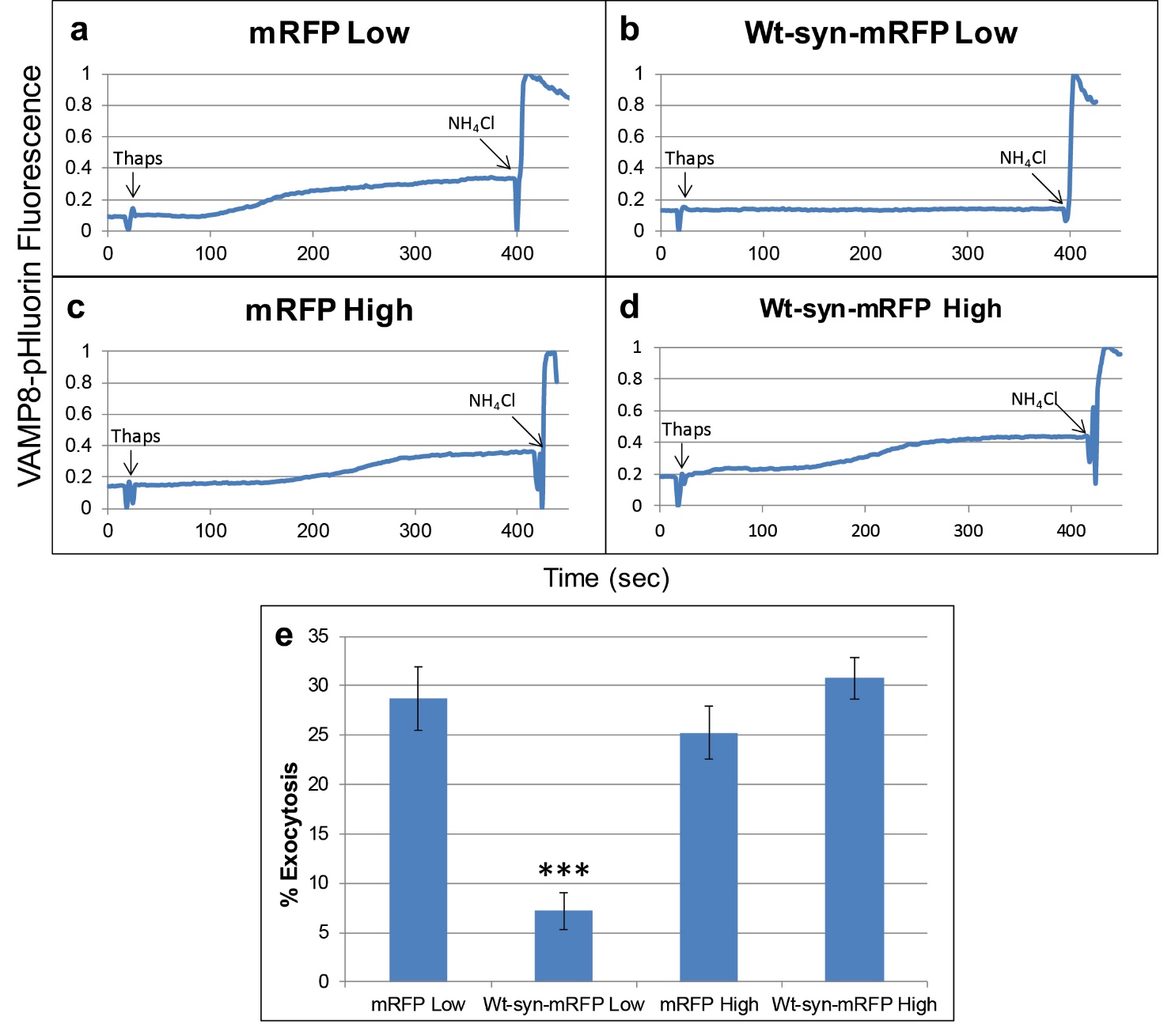


Supplementary Figure 7. **Low, but not high, expression levels of Wt a-syn-mRFP inhibit stimulated exocytosis of REs.** RBL cells were co-transfected with VAMP8-pHluorin and low (5μg) (**a**, **b**) or high (25μg) (**c**, **d**) levels of mRFP (**a**, **c**) or Wt a-syn-mRFP (**b, d**). Exocytosis was stimulated by thapsigargin (250 nM), and after 400 sec NH_4_Cl (50mM) was added to dequench remaining intracellular VAMP8-pHluorin fluorescence. **a**-**d)** Representative traces of VAMP8-pHluorin fluorescence integrated from movies of multiple confocal fields of 5-6 cells. **e)** Averaged relative exocytosis for many cells (n=55) as represented in (a-d); *** indicates P-values <0.001.


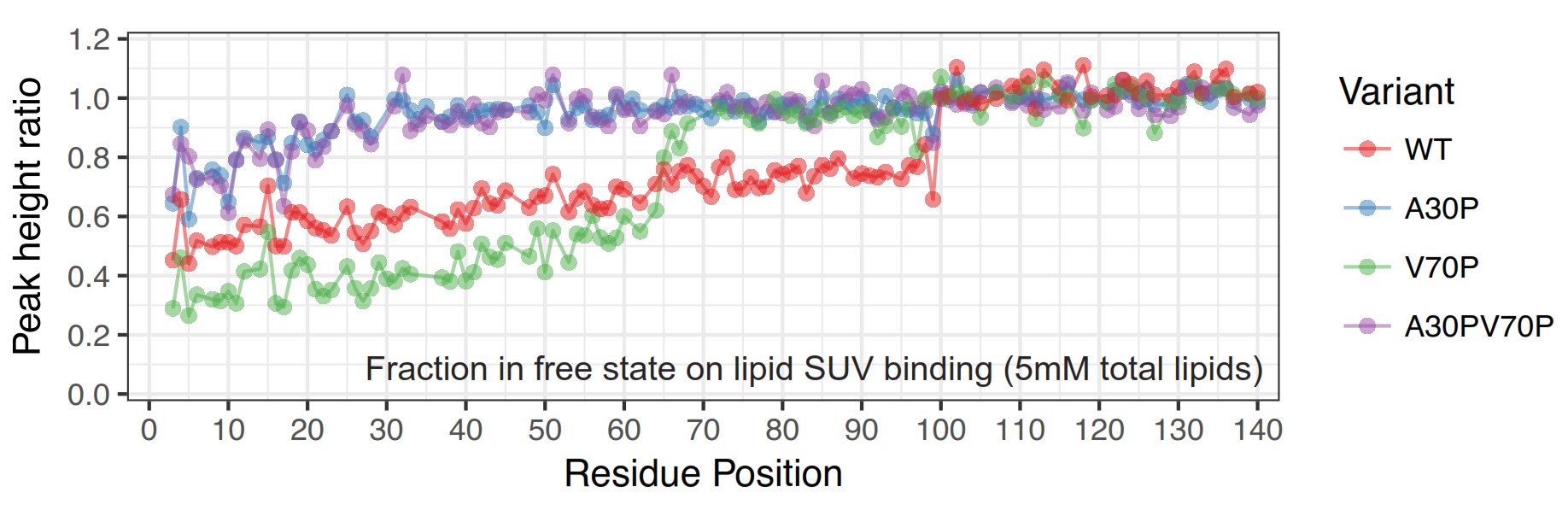


Supplementary Figure 8. **The A30P and V70P mutations result in release of a-syn regions C-terminal to the mutation site from vesicle membranes.** Vesicle binding of full-length a-syn variants measured as the ratio of NMR resonance intensities in the presence and absence of liposomes. The peak intensity ratio, representing the free fraction of each residue, is plotted for 50μM protein with small unilamellar vesicles (SUVs) containing 5mM total phospholipids at a molar ratio of DOPC:DOPE:DOPS = 60:25:15.


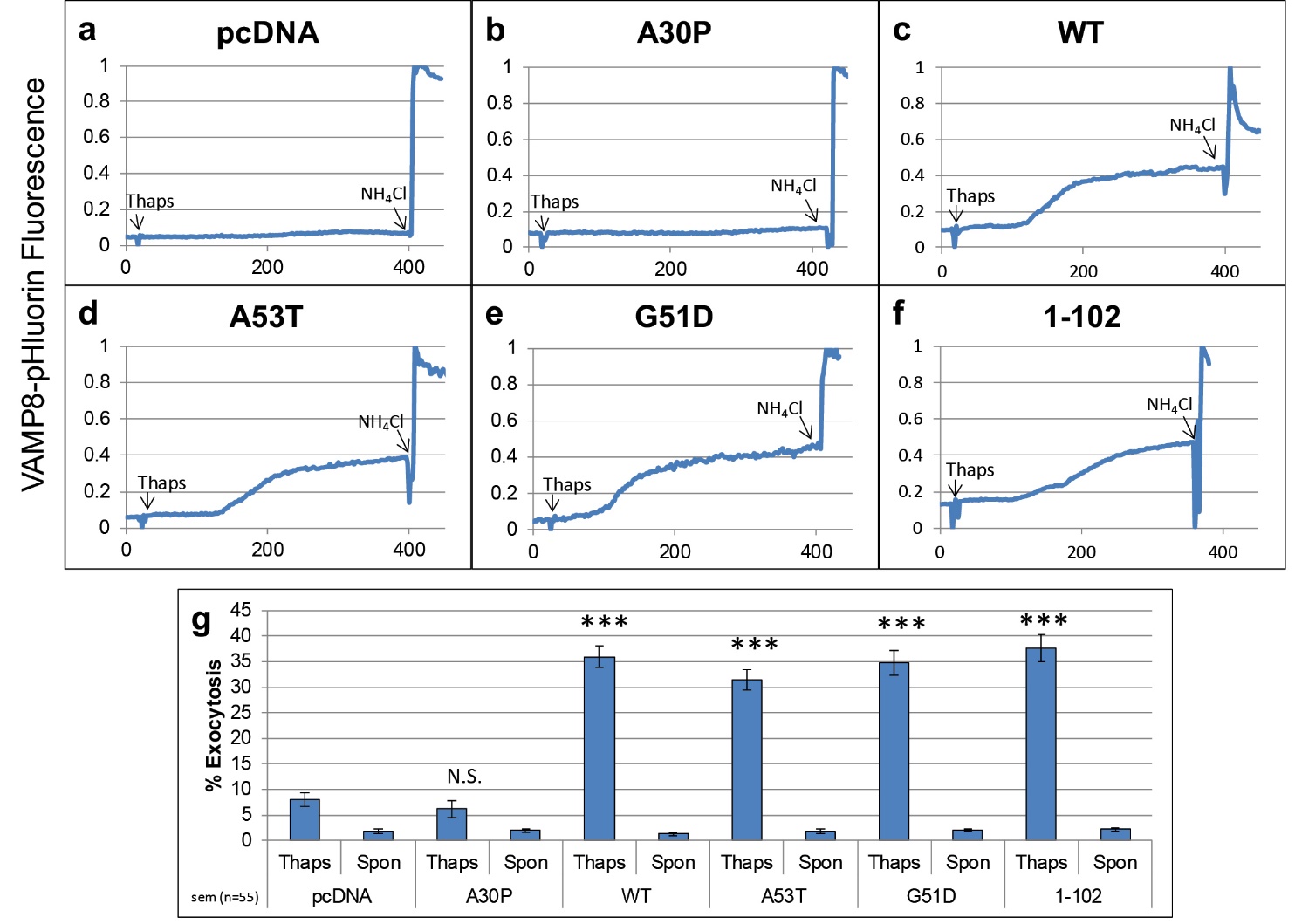


Supplementary Figure 9. **High expression of Wt a-syn and a-syn mutants except A30P enhance stimulated exocytosis.** RBL cells were co-transfected with VAMP8-pHluorin and high levels (25μg) of pcDNA (**a**) or A30P (**b**), Wt (**c**), A53T (**d**), G51D (**e**) or 1-102 (**f**) a-syn. Exocytosis was stimulated by addition of thapsigargin at t=20 sec, followed by addition of NH_4_Cl at ~t=400 sec. Representative traces showing average change in VAMP8-pHluorin fluorescence are integrated from multiple fields of 5-6 cells in confocal movies, similar to Supplementary Movies 2a,b. **g)** Summary from three independent experiments monitoring changes in VAMP8-pHluorin fluorescence in individual cells, before (spon) or plateauing after thapsigargin stimulation, normalized to fluorescence after addition of NH_4_Cl. Error bars are ± SEM for 55 individual cells for each condition; *** represents P-values <0.001, N.S. not statistically significant (P-value > 0.05).


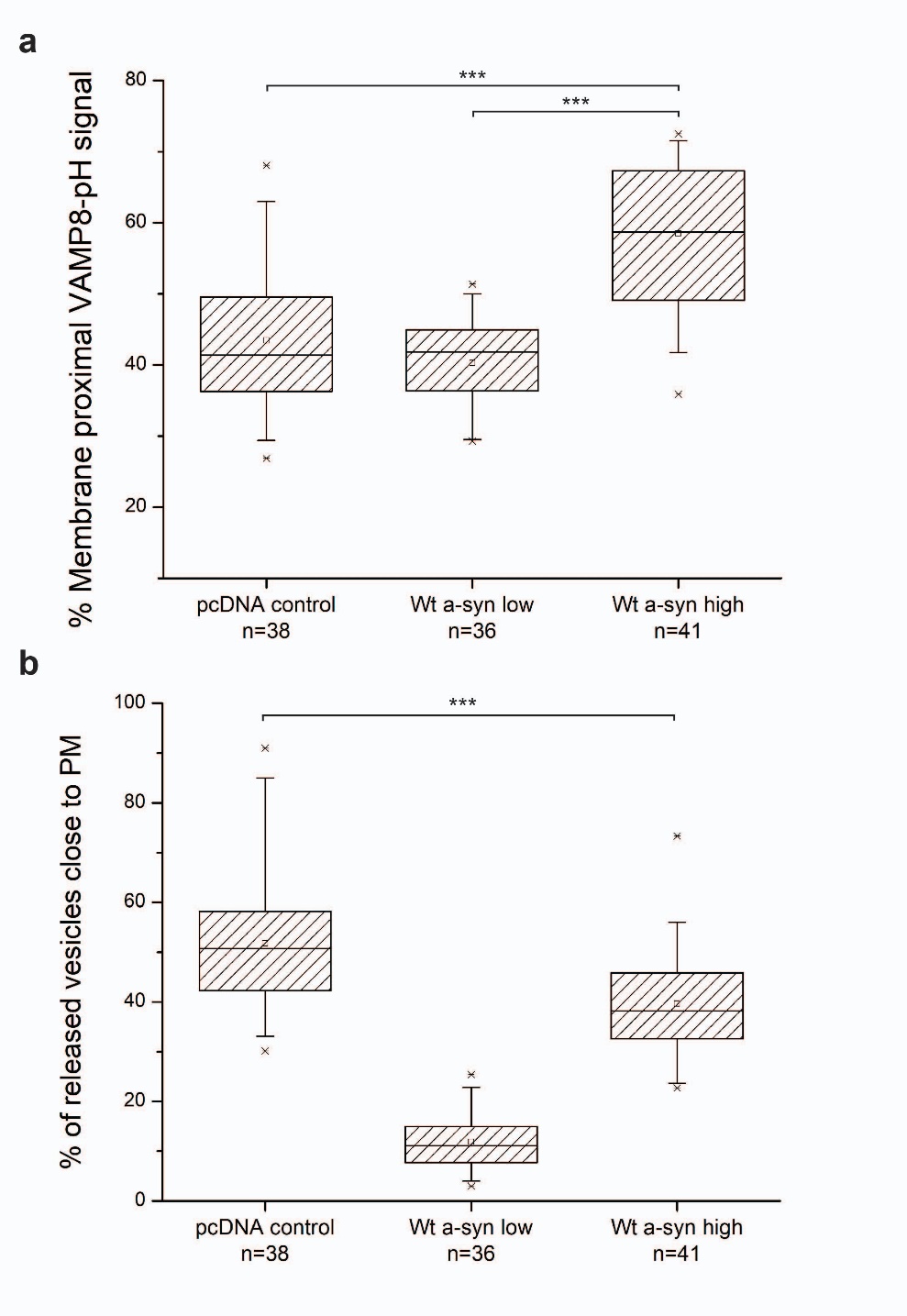


Supplementary Figure 10. **VAMP8-pHluorin fluorescence shows that high expression levels of Wt a-syn shift RE distribution to membrane proximal but that their exocytosis rate is less than controls.** RBL cells were co-transfected with VAMP8-pHluorin and pcDNA (control) or low (5μg) or high (25μg) levels of this plasmid containing Wt a-syn. VAMP8-pHluorin fluorescence was monitored in confocal movies (similar to Supplementary Movies 2a,b) before and after stimulation of exocytosis, and after addition of NH_4_Cl (300-400 sec after stimulation) to dequench intracellular VAMP8-pHluorin fluorescence. **a)** For samples of each type, a confocal image taken after the addition of NH_4_Cl was analyzed as for Figure 5: the fluorescence intensity within a thin layer around the plasma membrane (proximal region) was divided by total cell fluorescence to calculate % of total REs that are membrane proximal, representing both exocytosed and intracellular, non-exocytosed REs in this region. **b)** Two confocal images were compared for each sample: i) stimulated, before addition of NH_4_Cl and ii) stimulated, after addition of NH_4_Cl. Fluorescence in the thin layer, membrane proximal region (as described in part (a)) was measured for both images, and the fluorescence in this region measured *before* addition of NH_4_Cl (representing only exocytosed REs) was divided by the fluorescence *after* addition of NH_4_Cl (representing both exocytosed REs and intracellular, non-exocytosed REs proximal to the membrane). This calculation represents the fraction of membrane proximal REs that are released. For both (a) and (b) averaged values for each sample type (n= number of cells evaluated) are shown in a box plot. The box represents 25^th^-75^th^ percentile of the data, the midline represents the median and the small square represents the average. *** represents P-values <0.001.

**
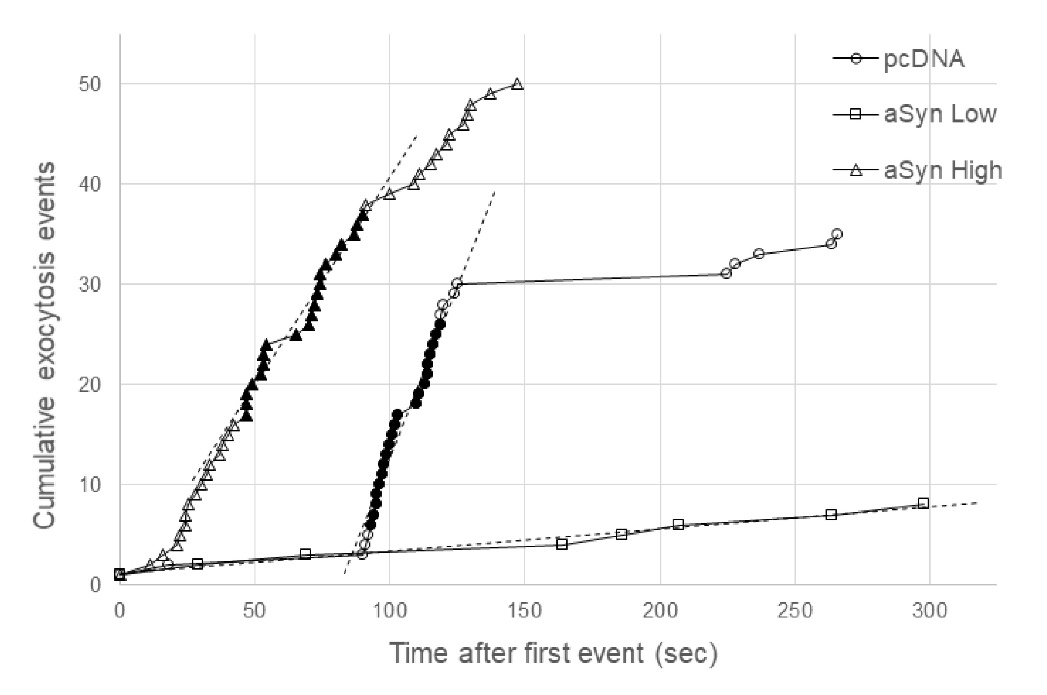
**

Supplementary Figure 11. **Exocytotic events counted in TIRFM movies show that lower Wt a-syn expression levels results in a lower level and rate of exocytosis, while higher Wt a-syn expression levels results in a higher level of exocytosis but at a reduced rate, compared to controls.** Cumulative exocytosis events determined from TIRF movies (Figure 2a-c and Supplementary Movies 1a-c) are plotted. Exocytosis events, appearing as spreading flashes of fluorescence, occurring within a 10 µm squared area on the basal membrane were counted manually and plotted starting with the first detectable exocytosis event. For cells transfected with pcDNA and Wt a-syn at high levels (25μg), the rate of exocytosis is defined as the slope at the midpoint of the steeply rising phase in each condition, and calculated by linear regression using 10 data points above and below the midpoint (points used shown in solid markers and the regression result as a dashed line). For cell transfected with Wt a-syn at low level (5μg), all 8 exocytosis events were used to calculate the slope. The rates of exocytosis determined in this way for pcDNA, a-syn low expression and a-syn high expression are 0.68, 0.02 and 0.41 events/second, respectively.


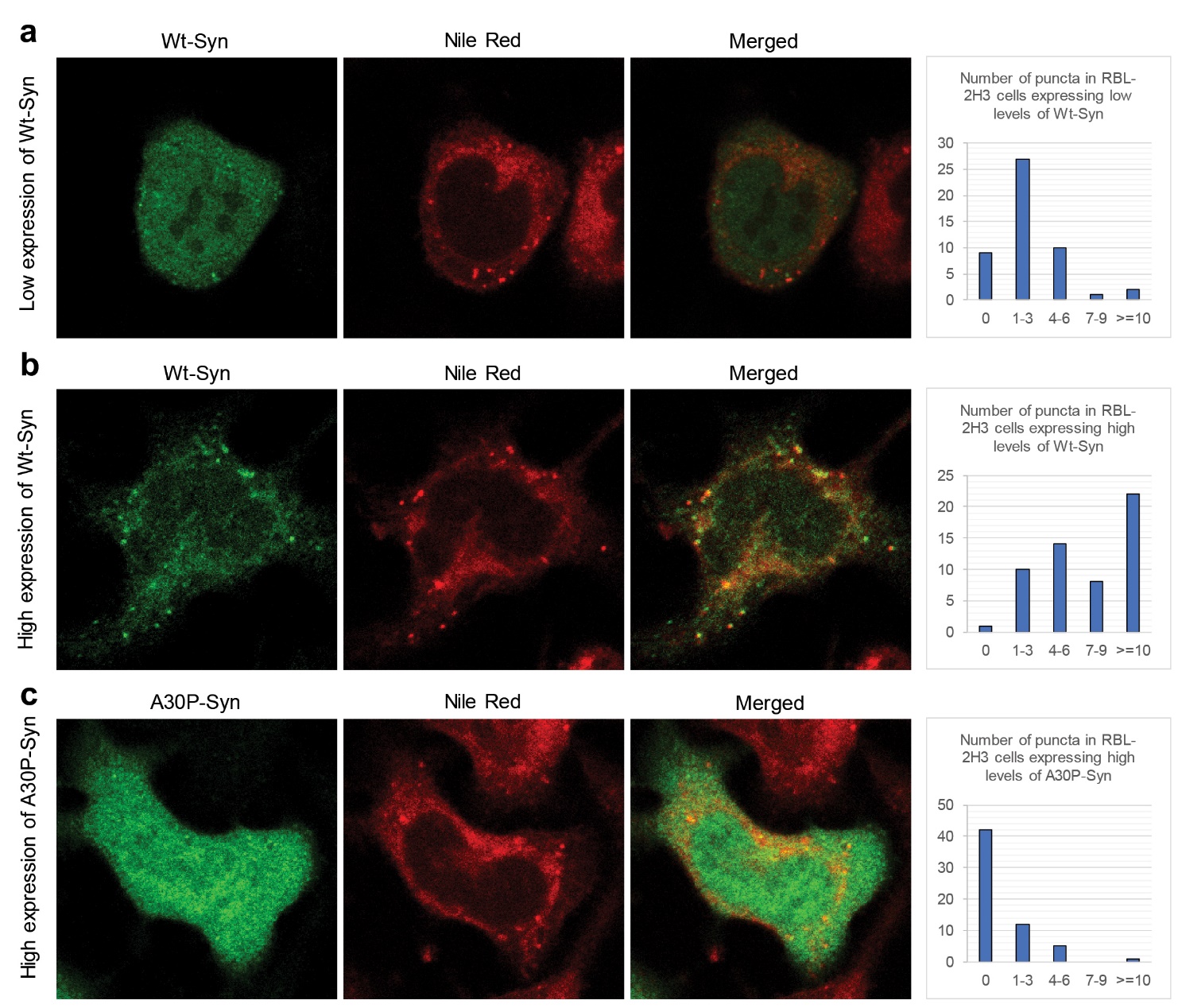


Supplementary Figure 12. **Wt but not A30P a-syn binds strongly to lipid droplets at high expression levels.** Confocal images of RBL cells transfected with low levels (5μg) of Wt a-syn **(a)** or high levels (25μg) of Wt a-syn **(b)** or A30P a-syn **(c)**. Cells were incubated with Nile red to label lipid droplets before fixing cells and immunostaining a-syn with Alexa-488. Histograms are derived from 3-D images of cells, and the number of lipid droplets coated with a-syn was quantified for 50 individual cells for each condition.


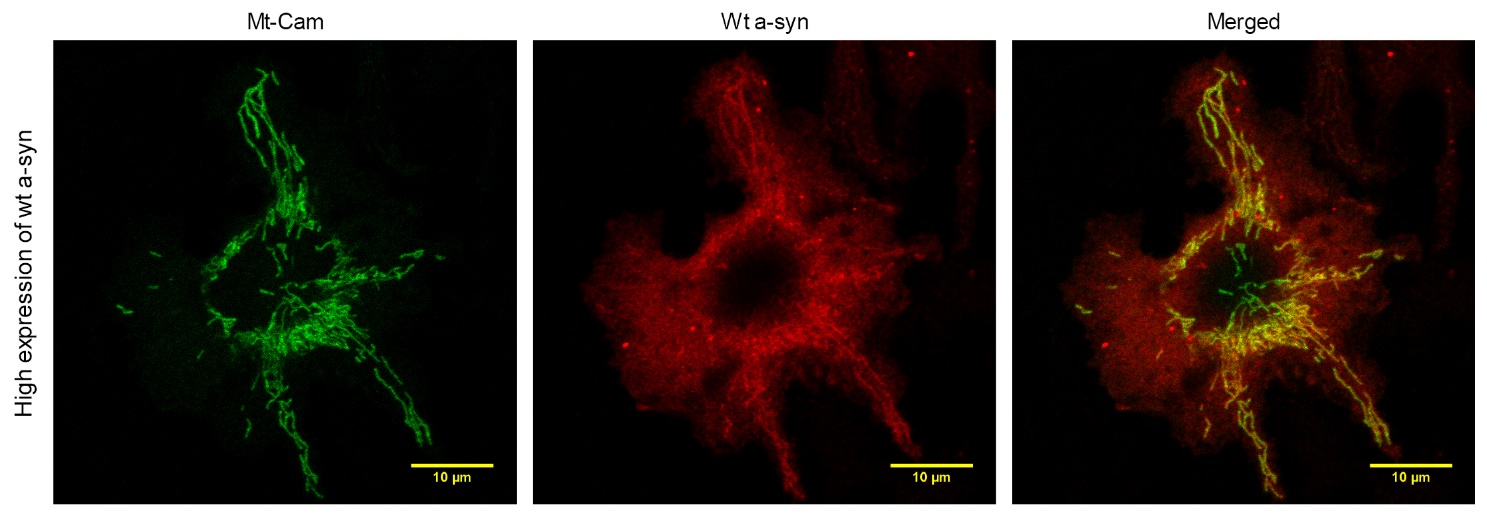


Supplementary Figure 13. **Wt a-syn at high expression levels binds significantly to mitochondria.** Representative confocal images of RBL cells co-expressing Mito-cameleon and high levels of Wt a-syn (immunostained with Alexa-568); scale bar = 10μm. Overlap of a-syn and mitochondrial labels for multiple cells under this condition is quantified and compared to samples with low/high levels of a-syn variants without and with mitochondrial stress in Figure 7f**.**

**Supplementary Movies Descriptions**

Supplementary Movies 1. **Low and high expression levels of Wt a-syn show differential effects on stimulated exocytosis of REs.** Representative movies were taken with TIRFM; scale bar = 10μm. RBL cells were co-transfected with VAMP8-pHluorin and empty plasmid pcDNA (**a**) or low (5 μg; (**b**)) or high (25 μg; (**c**)) levels of this plasmid containing Wt a-syn. Exocytosis was stimulated by thapsigargin, and VAMP8-pHluorin fluorescence increase was monitored before and after stimulation, and after addition of NH_4_Cl (50mM, 300-400 sec later) to dequench intracellular VAMP8-pHluorin fluorescence. Snapshots from these movies are shown in Figure 2.

Supplementary Movies 2. **Low expression levels of Wt a-syn inhibit stimulated exocytosis of REs.** Representative movies were taken with confocal microscopy; scale bar = 20μm. RBL cells co-transfected with VAMP8-pHluorin and low levels (5 μg) of pcDNA (**a**) or Wt a-syn (**b**) were sensitized with anti-DNP IgE and stimulated with 1 ng/ml DNP-BSA at 20 sec; 50 mM NH_4_Cl was added at ~300 sec to neutralize the intracellular spaces and dequench all VAMP8-pHluorin fluorescence. Snapshots from these movies are shown in Supplementary Figure 3.
